# Altered precipitation and root herbivory affect the productivity and composition of a mesic grassland

**DOI:** 10.1186/s12862-021-01871-0

**Published:** 2021-07-15

**Authors:** Kirk L. Barnett, Scott N. Johnson, Sarah L. Facey, Eleanor V. J. Gibson-Forty, Raul Ochoa-Hueso, Sally A. Power

**Affiliations:** 1 Hawkesbury Institute for the Environment, Western Sydney University, Locked Bag 1797, Penrith, NSW 2751 Australia; 2 Department of Biology, University of Cádiz, Avenida República Árabe Saharaui, 11510 Puerto Real, Cádiz, Spain

**Keywords:** C_3_:C_4_ ratios, Climate change, Community ecology, Rainfall regime, Root herbivores

## Abstract

**Background:**

Climate change models predict changes in the amount, frequency and seasonality of precipitation events, all of which have the potential to affect the structure and function of grassland ecosystems. While previous studies have examined plant or herbivore responses to these perturbations, few have examined their interactions; even fewer have included belowground herbivores. Given the ecological, economic and biodiversity value of grasslands, and their importance globally for carbon storage and agriculture, this is an important knowledge gap. To address this, we conducted a precipitation manipulation experiment in a former mesic pasture grassland comprising a mixture of C_4_ grasses and C_3_ grasses and forbs, in southeast Australia. Rainfall treatments included a control [ambient], reduced amount [50% ambient] and reduced frequency [ambient rainfall withheld for three weeks, then applied as a single deluge event] manipulations, to simulate predicted changes in both the size and frequency of future rainfall events. In addition, half of all experimental plots were inoculated with adult root herbivores (Scarabaeidae beetles).

**Results:**

We found strong seasonal dependence in plant community responses to both rainfall and root herbivore treatments. The largest effects were seen in the cool season with lower productivity, cover and diversity in rainfall-manipulated plots, while root herbivore inoculation increased the relative abundance of C_3_, compared to C_4_, plants.

**Conclusions:**

This study highlights the importance of considering not only the seasonality of plant responses to altered rainfall, but also the important role of interactions between abiotic and biotic drivers of vegetation change when evaluating ecosystem-level responses to future shifts in climatic conditions.

**Supplementary Information:**

The online version contains supplementary material available at 10.1186/s12862-021-01871-0.

## Background

Grasslands cover more than 40% of the global non-glaciated land surface [1] and are of high economic and ecological importance [2, 3]. However, many grassland systems exist in seasonal states of water limitation, and can be highly sensitive to changes in water availability [4, 5]. Furthermore, climate model predictions of shifts in the overall amount, timing and seasonality of rain events [6] are expected to result in prolonged and more intense droughts which, together with warmer temperatures and more frequent heatwaves, will reduce water availability and increase evaporative demand [7]. Such changes are likely to affect primary productivity and drive shifts in the community composition and species interactions within grassland ecosystems [8]. For example, it is generally understood that long-term increases in precipitation will lead to an increase in species richness, while decreases in precipitation will lead to the inverse; however, patterns of alternating wet and dry periods might, in the short-term, lead to higher rates of turnover and, hence, richness [9]. These relationships are further complicated by plant phenology whereby summer-active (C_4_) species may respond differently to altered soil water availability compared to those (predominantly C_3_) species that are active during the cooler seasons [10].

Invertebrates can be important components of grassland systems, although relatively little is known about their direct and indirect responses to precipitation changes [11, 12]. Drought and increased temperatures have both been shown to reduce time between insect generations, and increase the production of offspring, potentially leading to outbreaks in insect populations [13, 14]. While the majority of belowground invertebrate orders are non-herbivorous, some taxa are known to damage plant roots, resulting in significant impacts on plant productivity [15, 16]. In grasslands, for instance, up to a quarter of plant productivity can be lost to root herbivory [17], with the associated root damage having similar effects on plant performance as drought [18]. Furthermore, root damage can exacerbate the effects of soil moisture stress, further reducing water and nutrient uptake, directly influencing plant species competition [19, 20]. However, not all plants species are likely to be equally affected by root herbivores [21]. For example, in line with the C_3_–C_4_ preference hypothesis proposed by Caswell et al. (1973) [22], some root feeding scarabs (e.g. *Sericesthis nigrolineata*) favour C_3_ plants [23] whereas others (e.g. *Heteronychus arator*) favour C_4_ plants [24]. Furthermore, species- and functional group differences in plant responses to changes in rainfall patterns [25] could be compounded by varying levels of root damage due to root herbivores. In order to better predict plant community responses to climatic change, it is important to understand how belowground herbivory can modify these responses.

To this end, a large scale rainfall manipulation experiment was established in South East Australia, using rainfall shelters described by Power et al. (2016) [26]. We set out to characterise the short- to medium-term effects of three years of altered rainfall patterns (50% decrease in rainfall and reduced rainfall frequency) in a mesic, former pasture grassland, by quantifying effects on the productivity and composition of the plant community. A 50% reduction in rainfall amount was selected as the upper end of predicted changes in rainfall for Australia [27, 28], as well as representing the magnitude of annual rainfall, relative to the long term mean, for the 5 driest years in the past 100 years, based on local site data. In order to evaluate the role of root herbivory in ecosystem responses to altered rainfall, we specifically included a root herbivore addition treatment, in a factorial combination with these rainfall manipulations. We hypothesized that: (1) aboveground plant productivity and cover are decreased by both reduced amount and reduced frequency of rainfall inputs and that the magnitude of reduction is greater during the period of strongest growth. Additionally, plant community diversity is increased and evenness decreased by more variable (i.e. reduced frequency) rainfall, due to higher turnover rates and abundance of uncommon species [4]; (2) root herbivore addition reduces productivity and exacerbates the effects of drought on plant performance, via root damage [29]; (3) the ratio of C_3_:C_4_ plant functional types decline the most under the combined stressors of root herbivory and rainfall reduction [22, 25].

## Results

**Fig. 1:**
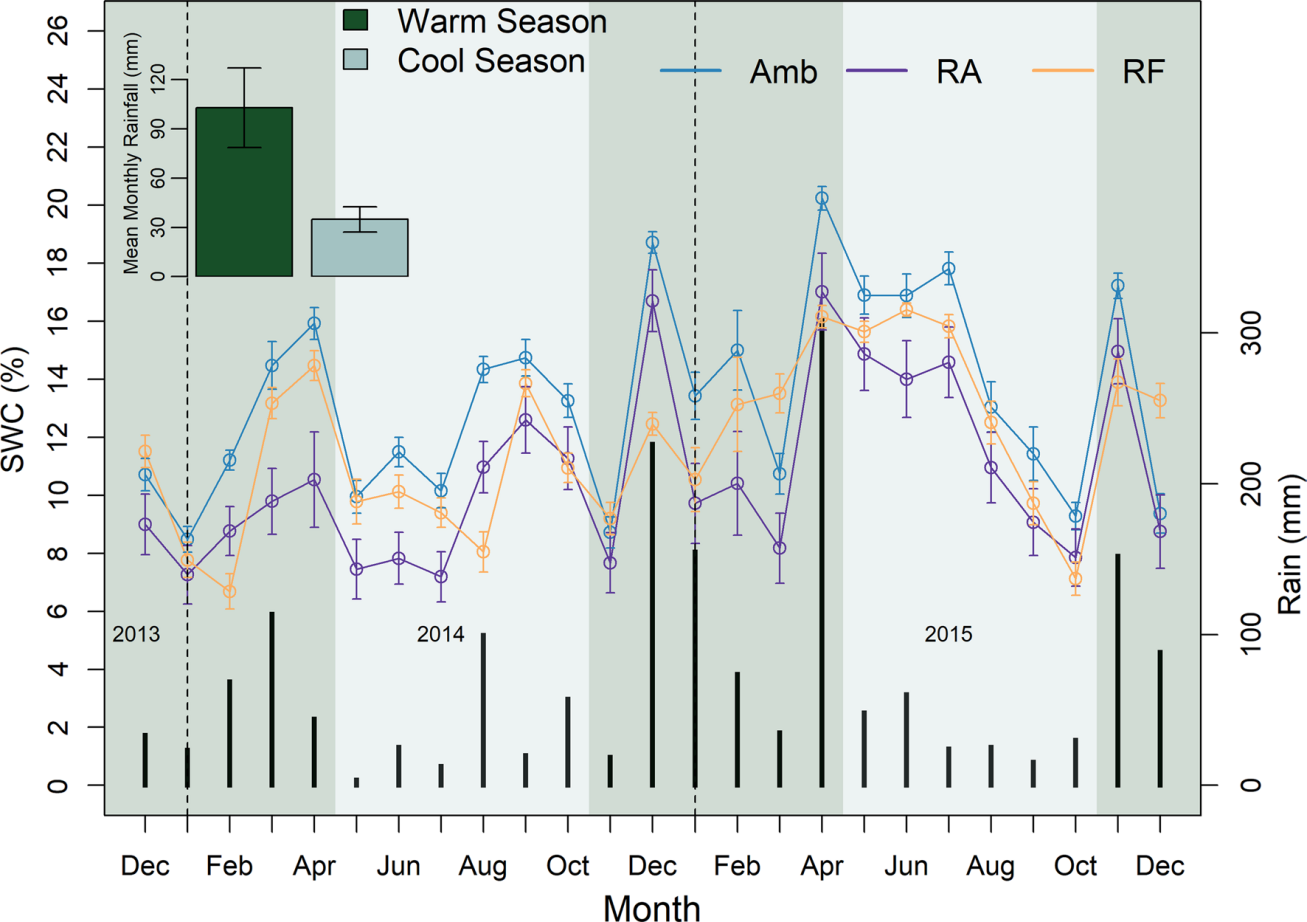
Seasonal mean Soil Water Content (%) (± SE) as recorded by automated soil moisture sensors buried (0–15 cm) in a subset of plots; error bars represent ± 1 standard error. Cumulative rainfall (mm), measured on site, is displayed across the bottom of the graph. The inset in the upper left displays the mean monthly rainfall per season; error bars represent ± 1 standard error

Volumetric soil water content (SWC) differed significantly between seasons (*χ*^2^ = 32.3, *p* < 0.001; warm season > cool season), as well as between rainfall treatment (*χ*^2^ = 6.42, *p* = 0.04; Amb & RF > RA). Amb and RF plots had higher average SWC than RA plots (Fig. 1). The coefficient of variation (CV) (stdev/mean) of weekly SWC indicated that RF rainfall had the highest variation in soil moisture in both seasons (cool: + 14%, warm: + 16%, compared to Amb); plots under RF rainfall also had the most weeks below 7% and above 15% SWC (Additional file 1: Fig. A1). Samples from the September 2015 soil excavation revealed a greater number of root-feeding scarabs in plots that had received root herbivore additions (6 ± 3 individuals in RH_0_ plots versus 23 ± 10 for RH_+_ plots [mean ± se], χ^2^ = 15.001, *p* < 0.001) (Additional file 1: Fig. A2, Table A49).

### Productivity & composition

#### Plant cover

**Fig. 2:**
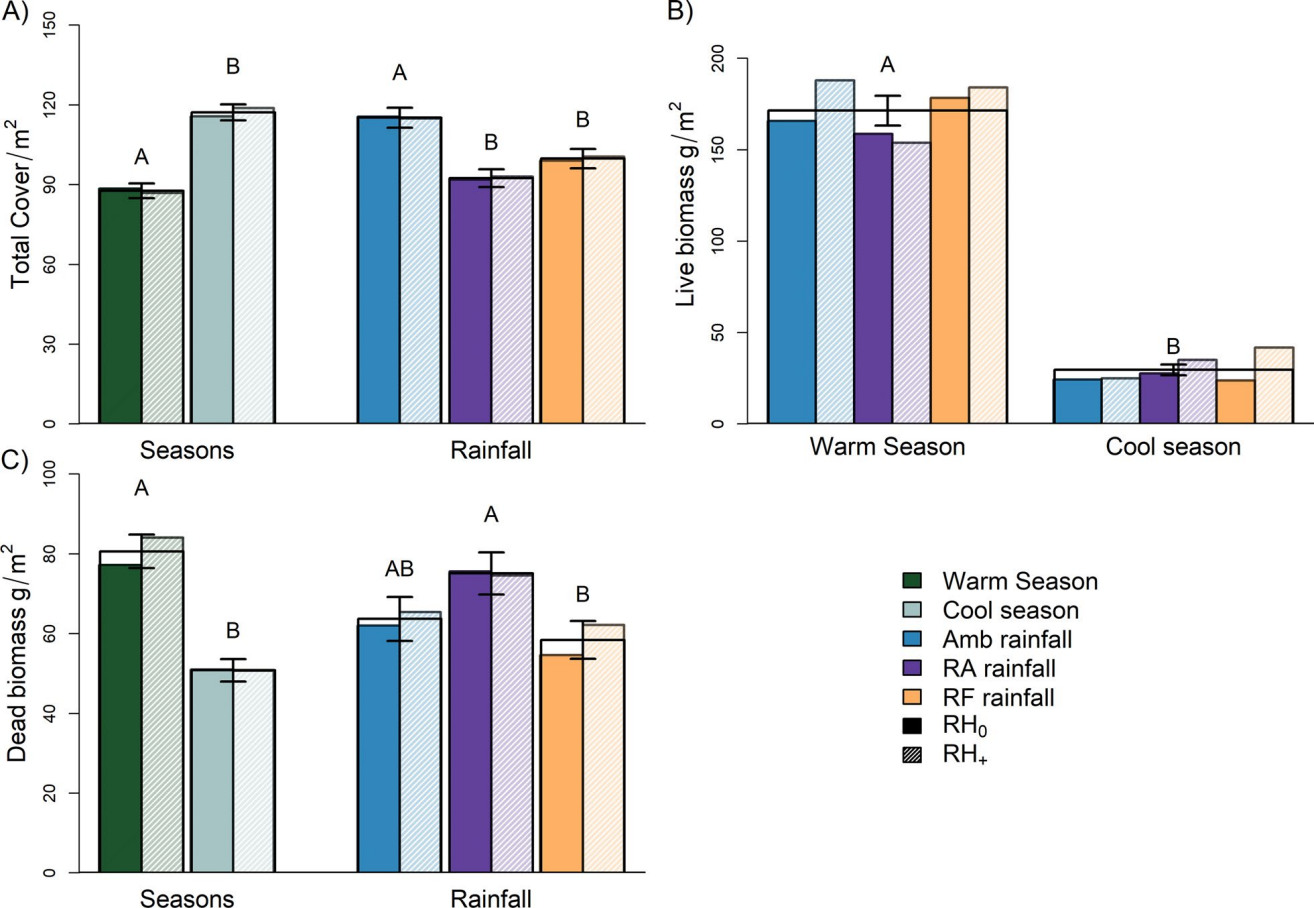
Plant responses to rainfall treatments: **A** total plant cover grouped by season and by rainfall, **B** total live biomass grouped by rainfall treatment per season and **C** total dead biomass grouped by season and by rainfall treatment. Fill for bars corresponds to rainfall treatments (Amb: ambient rainfall, RA: 50% reduced amount rainfall, RF: reduced frequency rainfall) and seasons. Opaque fill and hatching correspond to root herbivore status (RH_+_: root herbivores added, RH_0_: root herbivores not added); error bars correspond to standard ± 1 error. Letters indicate post hoc comparisons without a herbivore treatment distinction

We found significantly greater plant cover (+ 37%) in the cool season compared to the warm season (*F*_1,60_ = 65.9, *p* = 0.001, Additional file 1: Table A1). *Paspalum* spp. (C_4_) was the most prevalent species in both seasons, with *Microlaena stipoides* (C_3_) also common across plots in both seasons. However, many C_3_ species (e.g. *Vicia sativa* and *Lolium perenne*) had high cover in the cool season and died back in summer/autumn while C_4_ grasses (e.g. *Cynodon dactylon* and *Setaria parviflora*), had higher cover in the warm season and much lower cover in winter/spring. There was also a significant effect of rainfall (*F*_2,60_ = 13.6, *p* < 0.001, Additional file 1: Table A2) with both RA and RF treatments having lower plant cover than Amb plots (Fig. 2A). Analysis of pre-root herbivore addition (i.e. baseline) plant data showed no significant differences in cover for rainfall or root herbivore addition treatments and no treatment interactions.

#### Aboveground plant biomass

Overall rainfall treatment effects for total (dead + live) and live biomass, across all sample periods, were not significant. There was significantly higher total (dead + live) (*F*_1,60_ = 249, *p* < 0.001) and live (*F*_1,60_ = 282, *p* < 0.001) biomass in the warm season (November–April) compared to the cool season (May–October) (Fig. 2B). We also found significantly more standing dead biomass in the warm season compared to the cool season (*F*_1,60_ = 35.7, *p* < 0.001) and in the RA compared to RF rainfall treatment plots (neither RA or RF were significantly different from Amb rainfall treatment plots) (*F*_2,60_ = 3.89, *p* = 0.017, Additional file 1: Tables A5–A17) (Fig. 2C). Analysis of pre-root herbivore addition (i.e. baseline) data showed significantly higher live biomass in herbivore added plots (*F*_1,30_ = 12.6, *p* < 0.001).

#### Root productivity

There was significantly lower productivity in RF compared to Amb and RA rainfall plots (*χ*^2^_2,36_ = 7.05, *p* = 0.029), but no effect of root herbivore addition. However, analysis of pre-root herbivore addition data (i.e. baseline) also showed significantly lower root mass in RF plots (*F*_2,30_ = 5.01, *p* = 0.013, Additional file 1: Tables A18, A19).

#### C_3_/C_4_ ratios

**Fig. 3:**
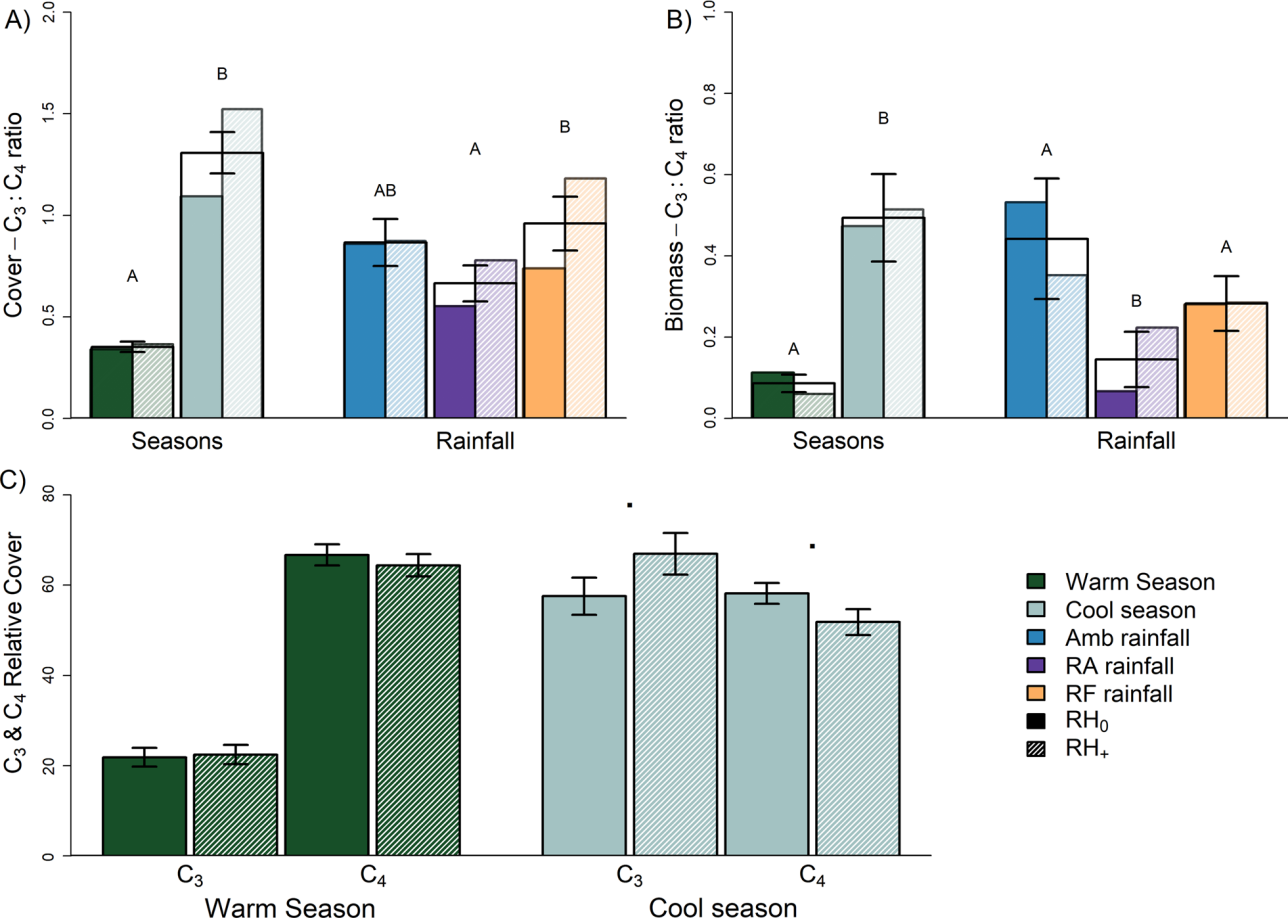
Plant responses to rainfall treatments: **A** C_3_:C_4_ plant cover ratio in response to season and rainfall, **B** total live (green) biomass C3:C4 ratio grouped by season and by rainfall and **C** total plant cover per plot across all species (C_3_ and C_4_), in both seasons and in both added and non-added herbivore plots. Fill for bars corresponds to rainfall treatments (Amb: ambient rainfall, RA: 50% reduced amount rainfall, RF: reduced frequency rainfall) and seasons. Opaque fill and hatching correspond to root herbivore status (RH_+_: root herbivores added, RH_0_: root herbivores not added). Error bars correspond to standard ± 1 error. Letters within panels **A** and **B** indicate post hoc comparisons without a herbivore treatment distinction. Within panel **C**, “**·**” indicates marginal significance between RH_0_ and RH_+_ plots within season

For both biomass and cover, there was a significant difference in C_3_:C_4_ ratios between seasons (cover: *F*_1,132_ = 133, *p* < 0.001, biomass: *F*_1,60_ = 50.8, *p* < 0.001; Fig. 3A, B). The cover-based C_3_/C_4_ ratio in the cool season was three times greater than in the warm season, and the biomass-based ratio was seven times higher. There was also a significant effect of rainfall on the biomass-derived C_3_:C_4_ ratio (*F*_2,60_ = 8.92, *p* = 0.002; Amb > RF > RA), and a significant effect of herbivore treatment (*F*_1,132_ = 4.71, *p* = 0.038) and rainfall (*F*_2,132_ = 3.58, *p* = 0.034) on the cover-derived ratio, as well as a marginal interaction between season and root herbivore addition (*F*_1,132_ = 2.91, *p* = 0.076, Fig. 3C, Additional file 1: Tables A20, A21). Root herbivore addition resulted in a marginally significant increase in the cover-based C_3_:C_4_ ratio in the cool season. Analysis of pre-root herbivore addition (i.e. baseline) data showed no significant differences in cover or biomass C_3_:C_4_ ratios for either treatment (rainfall and root herbivore addition).

### Plant diversity and evenness

**Fig. 4:**
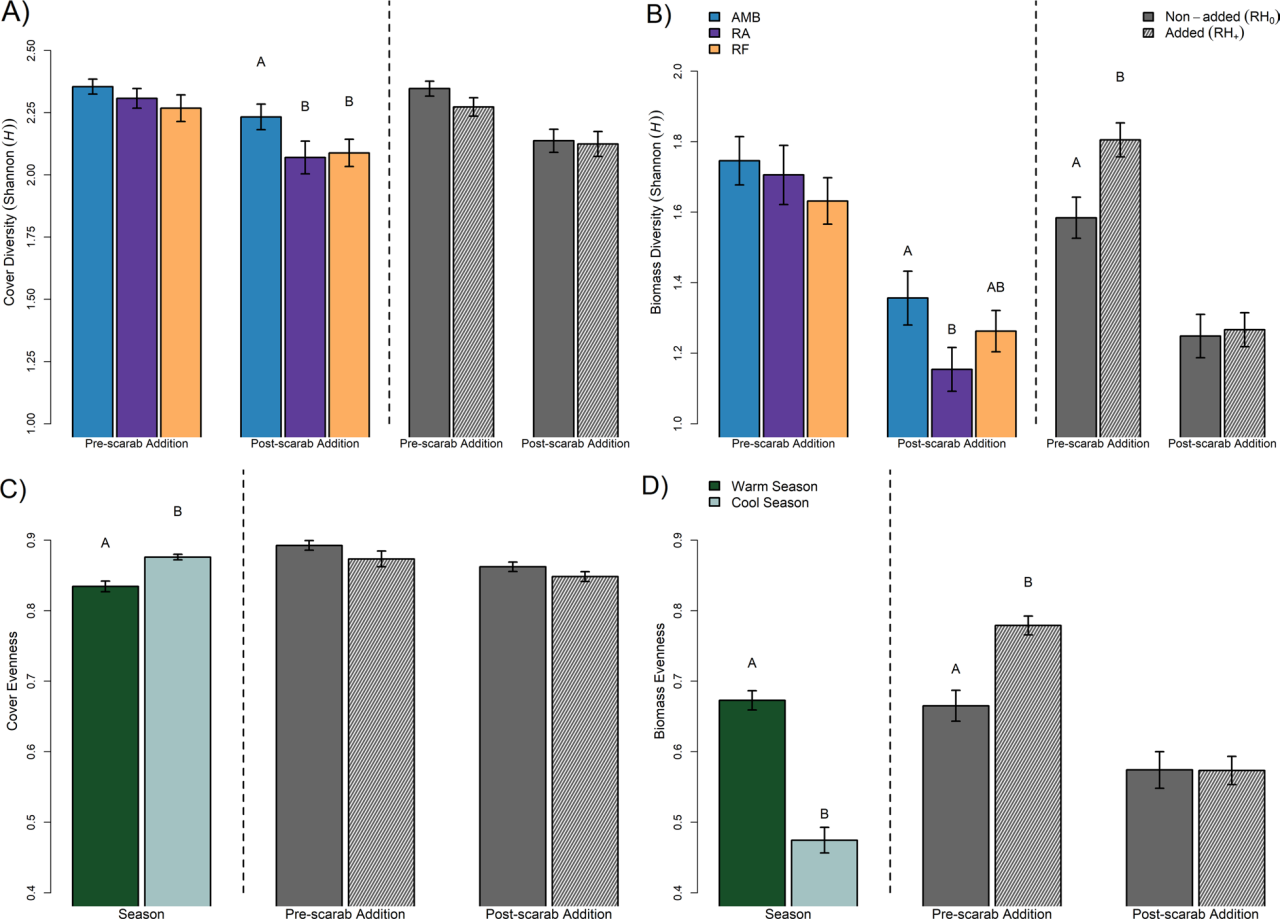
Rainfall treatment effects, pre- and post-root herbivore addition, on plant community diversity (Shannon's (*H*)) for **A** cover and **B** biomass. Remaining panels display seasonal effects and plant community evenness pre-/post-root herbivore addition for **C** cover and **D** biomass. Fill for bars correspond to rainfall (Amb: ambient rainfall, RA: 50% reduced amount rainfall, RF: reduced frequency rainfall) treatments and seasons, and hatching corresponds to root herbivore status (RH_+_: root herbivores added, RH_0_: root herbivores not added); error bars correspond to standard ± 1 error. Letters indicate post hoc comparisons

Biomass-based diversity (*Shannon–Wiener*) was significantly lower in RA and RF plots, compared to Amb (biomass: *F*_2,60_ = 3.43, *p* = 0.037), while cover-based diversity was lower in RA, compared to Amb and RF (cover: *F*_2,60_ = 6.78, *p* = 0.003). There was also significantly higher cover-based diversity in the cool season, compared with the warm season (*F*_1,60_ = 135, *p* < 0.001), and the opposite pattern was apparent for biomass-based diversity (higher in the warm season, compared with the cool season (*F*_1,60_ = 50.2, *p* < 0.001) (Fig. 4A, B). Plant community evenness measures showed similar patterns, with biomass-based evenness significantly lower in the cool season compared to warm (*F*_1,60_ = 78.0, *p* < 0.001) and the opposite pattern was evident for cover-based evenness (*F*_1,60_ = 24.1, *p* < 0.001, Additional file 1: Table A23, A25). Analysis of pre-root herbivore addition data showed significantly higher diversity (*F*_1,30_ = 8.35, *p* = 0.010) and evenness (*F*_1,30_ = 19.3, *p* < 0.001) for biomass-based data in RH_+_ plots (Fig. 4C, D).

## Discussion

Overall, we found strong seasonal dependence in plant community responses to both rainfall and root herbivory. Rainfall reduction (both in terms of amount and frequency) resulted in lower plant cover and diversity, which was most evident in the cool season. We also observed that the reduced rainfall amount treatment decreased the relative biomass of C_3_ compared to C_4_ plants, and root herbivore addition tended to increase C_3_ cover and decrease C_4_ cover, but only in the cool season.

### Soil water content is affected to differing extents by reductions in rainfall amount and frequency

Rainfall treatments had significant effects on soil moisture. Generally, ambient rainfall plots had the highest SWC (13.5%), followed by those receiving the RF (11.8%) and then RA (10.8%) treatments. It is important to note, however, that RF plots experienced greater variability in soil moisture, while RA and Amb plots had about the same number of weeks < 7% and > 15% SWC. This is similar to findings from other mesic grassland systems where reducing the frequency of rainfall events without reducing total rainfall amount both decreased average SWC and increased its variability [30: − 8%, 31: − 19%, 32: ca. − 3%].

#### Prediction 1)

Aboveground plant productivity and cover are decreased by both reduced amount and reduced frequency of rainfall inputs and the magnitude of reduction is greater during the period of strongest growth. Additionally, plant community diversity is increased and evenness decreased by more variable rainfall (i.e. reduced frequency)

Both reductions in the amount and frequency of rainfall reduced total plant cover, although productivity was unaffected. This discrepancy may reflect differences in the timing of cover and biomass sampling in relation to seedling recruitment and/or life history strategies (i.e. short reproductive cycles, early/late vegetative phenology). While contemporaneous measurements of cover and biomass typically show a linear relationship [30], differences in the timing of rainfall or the dominance of certain species at different times of the year can shift this relationship when cover and biomass are not recorded at the same time [31, 32]. For example, annuals (mostly C_3_ species in this instance) are thought to be better exploiters of disturbance (i.e. biomass removal during twice-yearly harvests) because they are assumed to invest more resources in leaf and seed production rather than roots [33, 34]. At the same time, perennials are expected to compete more strongly for soil water and light [35, 36]. Here, the removal of standing biomass in twice-yearly harvests may have given annuals a temporary advantage in the early re-growth part of both cool and warm seasons, coinciding with cover sampling efforts, while end of season harvests reflected perennial dominance due to their higher competitiveness and persistance, relative to the shorter life cycle of annuals. This could also explain the lack of rainfall effects on biomass since communities may have stabilized by the time biomass sampling was undertaken. At the same time C_3_ annuals (especially legumes) are expected to be more sensitive to rainfall [37, 38] and, therefore, cover estimates recorded at a time when they were dominant could have resulted in an early season rainfall effect for cover but not for later measurements of biomass production, when such species were likely to have died back.

Although seasonal differences in both cover and productivity were highly significant we did not see stronger effects of rainfall treatments in the warm (main growing) season, contrary to our prediction. Research elsewhere, in semi-arid communities, has shown that the presence of an invasive exotic plant can attenuate responses of native grasses and forbs to variable rainfall associated with seasonal rainfall shifts [39]. The current study took place in an old field, with several exotic species that have been reported to become invasive outside of managed pasture systems [40, 41]. These species may have suppressed any strong rainfall responses by less invasive exotics or native grasses and forbs during the period of strongest plant growth (i.e. warm season), making it more difficult to detect treatment effects. Indeed, *Cynodon dactylon*, an exotic naturalised grass, produced relatively more biomass under RA rainfall compared to Amb and RF, whereas other species, such as the native *M. stipoides*, were reduced under RA and RF rainfall (Additional file 1: Table A50).

The greater amount of standing dead plant material in plots under the RA rainfall treatment, compared to ambient and RF, likely reflects a lower overall soil water content in this treatment, coupled with longer periods of particularly low soil moisture [42]. Given the lower relative proportion of live/green C_3_ plant material (compared to C_4_ species) in biomass harvests for RA plots, the greater quantity of standing dead biomass in this treatment may reflect tissue senescence/death of the less drought-tolerant C_3_ species, particularly when exposed to periods of very high temperatures that occur frequently during the warm season at our field site. C_4_ plants are expected to be more tolerant to drought due to their inherently higher water use efficiency [43] and were generally more abundant in plots receiving a reduced amount of rainfall, compared to RF and ambient plots. The greater density of dead material in RA plots could, however, reduce evaporative water loss from the soil surface in these treatments [44, 45]. Furthermore, since C_3_ grasses frequently have higher transpiration rates than C_4_ grasses [46, 47], a shift towards C_4_ dominance in these plots could also reduce transpirational water loss, potentially buffering treatment effects on soil water content.

In contrast to aboveground measures, we found that reductions in the frequency of rainfall events significantly reduced root productivity, which contrasts with findings from Fay et al. [30]. They reported increased root biomass in a Kansas C_4_ dominated mesic tallgrass prairie. It is possible that the sampling design in our study, restricting productivity measurements to the top 0–10 cm, may have failed to capture species’ shifts to deeper rooting distributions in response to rainfall treatments, a finding that has been reported for grass species in other rainfall manipulation studies [49, 50]. Indeed, several of the species in this study have been reported to have rooting depths up to 1.24 m, depending on the site [51].

Biomass and cover-based diversity and evenness estimates were strongly diminished by reductions in the amount and frequency of rainfall, which is contrary to our prediction. Knapp et al. [4], for example, found that increases in precipitation variability increased diversity and evenness. This difference could be related to the relatively high mean warm season soil water content recorded in the grassland they studied, which had a range of 20–45% [4]. Our DRI-Grass site, in contrast, ranged from 5 to 24% during the wettest and warmest part of the year (December–March), possibly indicating that our grassland experiences a higher degree of water limitation due to high summer temperatures and well-drained soils. Treatment perturbations on top of this level of water stress likely resulted in reduced species establishment, as well as seasonal loss of species. Contrasting treatment effects on diversity between our study and that by Knapp et al. [4] may also reflect differences in starting levels of species richness and plant density specific to each study area. For example, some species-rich plant communities have the potential to suffer more in extreme weather as a result of higher density-dependent water consumption [52, 53]. Moreover, the method of sampling the plant community can have a drastic effect on the estimated plant diversity and ANPP, making comparisons between this study and others challenging [54, 55]. Here we removed not only live biomass but also standing dead (litter), most likely altering the nutrient dynamics and microenvironmental variables of the community [56, 57]. Lastly, the short time-frame of the study (ca. 2 years) may not have captured community shifts in response to alterations in rainfall, as it can take several years, depending on the species pool, to see natural immigration significantly impact the plant community [9]. The linkages between productivity, diversity and rainfall in grasslands will undoubtedly also rely heavily on time scale, precipitation history, soil type and the underlying traits of the plant community [58].

#### Prediction 2)

Root herbivore addition reduces productivity and exacerbates the effects of drought on plant performance via root damage

We found very little evidence for effects of root herbivore addition on grassland community productivity. However, pre-existing differences in plot biomass prior to commencement of the herbivore addition treatment did diminish and became non-significant over time. We still did see greater biomass in those same plant communities over the full 2 year study period, possibly indicating some level of compensation in the face of increased root-herbivore density. In fact, just a history of root herbivore activity can alter plant community productivity. For example, Sonnemann et al. [61] found that soil trained with root herbivores caused plant communities in those same soils to be significantly more productive. This was most likely due to changes in arbuscular mycorrhizal fungal communities and their effects on plant growth [59, 60]. In our case, changes in root herbivore activity could have resulted in an increase in beneficial soil biota [61] or an increase in metabolite production [62], offsetting any effects of increased root damage. Future studies examining soil scarab impacts should ideally time soil biota surveys to correspond with aboveground plant community surveys in order to better understand plant responses to climate-change induced stresses.

As for root herbivore additions exacerbating the effects of reduced rainfall, we found no support. It could be that our inoculation did not produce a density threshold of root herbivores that would have elicited detectable declines in plant production [63]. Indeed, field studies have found aboveground damage linked to scarab densities in the range of 20–60 individuals per m^2^ [64], while pot studies show that an average of 42 individuals per m^2^ [65] can cause declines in root and foliage biomass; we found a mean of 23 scarab larvae per m^2^ in RH_+_ plots when soil samples were assessed, ~ 18 months after initial inoculations took place. Whilst compensatory growth in response to root herbivory is expected to be unlikely [18], it is not absent from the literature [66, 67]. We could have also missed the effect of higher densities of root-feeders due to the short time-frame of the study, as delayed density-dependent effects on grasslands can take three or more generations to occur [68]. Equally as likely, the root herbivores used in this study could have been selectively feeding on species of higher nutritional quality, thereby releasing those of lower quality from competition [22].

#### Prediction 3)

The ratio of C_3_:C_4_ plant functional types decline the most under the combined stressors of root herbivory and rainfall reduction

While we predicted that the effects of rainfall reduction and root herbivory would be additive, we did not find evidence of this. As mentioned previously, the cover based C_3_:C_4_ ratio was marginally higher in plots that had root herbivores added, but only in the cool season. This was a result of both an increase in C_3_ cover and a reduction in C_4_ cover, relative to control plots. Root herbivores could be preferentially feeding on C_4_ plants rather than C_3_ plants, although this is contrary to expectations based on previous research [71, 72]. Data collected in a different study from the same site indicated that reductions in rainfall amounts may increase the C:N ratio in the roots of C_3_ forbs but decrease the ratio in roots of C_4_ grasses (K. L. Barnett unpublished data), potentially making the latter more prone to root herbivory [73]. Alternatively, C_4_ plants could have greater root biomass and, hence, would be attacked more often due to a high encounter rate by belowground herbivores [30]. Two of the most abundant C_4_ species at the site (*C. dactylon* and *P. dilatatum*) were shown to have greater root biomass than two co-occurring C_3_ species (*M. stipoides* and *L. perenne*) in a parallel, pot-based study [52]. While the biomass C_3_:C_4_ ratio was reduced by rainfall treatments, especially when subjected to reduced rainfall amount, root herbivore addition did not modify this response in any way. The relatively greater sensitivity of C_3_ plants to mean annual precipitation, compared to C_4_ grasses, is in line with the wider literature [74].

## Conclusions

In this study, we found that rainfall had a much stronger influence on grassland productivity and plant community composition than root herbivory. No interaction between root herbivore addition and reductions in either the amount or frequency of rainfall was apparent, but root herbivore effects, while subtle, contributed to increased cool season dominance of C_3_ plants. Reduced amount of rainfall in particular shifted the plant community towards C_4_ species dominance. Ultimately, we found evidence that both root herbivores and variable rainfall, on their own, can impact grassland community productivity and composition, even with relatively small numbers of root feeders. Potentially larger impacts (and possibly interactions) might be seen when associated with large outbreaks of root-feeding insects, as is predicted under future climate change scenarios [24, 75]. The linkages between rainfall, herbivory and plant communities identified in this study clearly highlight the need to consider biotic (e.g. above- and belowground invertebrate communities [76]), alongside abiotic components when evaluating and predicting ecosystem-level responses to future climate change.

## Methods

### Experimental site

The DRI-Grass experiment (Drought and Root herbivore Impacts on Grassland) is located in a former mesic pasture grassland (33° 36′ 34.65″ S, 150° 44′ 18.39″ E) and has not had any nutrient input in the past 30 years. The soil is classified as an alluvial Blackendon Sand [77] and is a low-fertility sandy loam with low organic matter content (0.7%) and relatively low water holding capacity (~ 20% [78]). The climate in Richmond, New South Wales is classified as humid, subtropical—Group Cfa according to the Köppen-Geiger climate classification [79]. As such, both autumn/winter (cool season [May–October]: 13.7 °C, 297 mm) and spring/summer (warm season [November–April]: 21.5 °C, 508 mm [80]) temperature and rainfall levels are conducive to growth. The site had an annual average temperature of 17.0 °C and annual average rainfall of 861.6 mm during 2013–2015 (data collected on site obtained from automatic sensors [26]).

### Rainfall and root herbivore treatments

The DRI-Grass experiment consists of 48 plots (1.9 × 2.5 m) with fixed rainout shelters that exclude ambient rainfall inputs, plus 12 uncovered infrastructural control plots. The shelters have open sides to minimize microclimate changes, and are covered with clear, UV-transparent Perspex roofs sloped at an angle of 18°. Here, we used a sub-set of 36 sheltered plots representing three simulated rainfall regimes. Rainfall treatments included: ambient (Amb: rainfall applied equal to that measured on site in a 24 h period), reduced amount (RA: 50% of ambient rainfall), and reduced frequency (RF: 21 days without water, followed by a single application of accumulated rainfall that occurred during that period). These watering treatments were based on model predictions of reduced rainfall amount and, in particular, reduced frequency of rainfall events by mid-century in many parts of SE Australia [81]. The RA treatment also represents the magnitude of annual rainfall reduction, relative to the long term mean, for the 5 driest years in the past 100 years, based on local site data [27, 28]. All three rainfall treatments (Amb, RA and RF) had 12 replicates, six with root-feeding scarabs (see below) added and six with background levels of scarabs. Treatments were randomised within six replicate blocks. Shelters were constructed facing into the direction of the prevailing wind, to minimise ingress of natural rainfall. An impermeable root barrier was installed around each plot to a depth of 30 cm, preventing adjacent water flow and root incursion. A tipping bucket rain sensor (0.2 mm graduation, ICT International, Armidale, NSW, Australia) was used to automatically log the amount of rainfall in a 24 h period, which was then added to the plots according to treatments, during non-daylight hours. Soil moisture TDR probes (CS616, Campbell Scientific, Thuringowa, QLD, Australia) were used to measure soil moisture in the top 15 cm of the soil profile (n = 3 per treatment combination). Water addition was achieved through a calibrated flow meter connected to four height-adjustable, 90° spray heads located in each corner of the plot. Rainfall treatments commenced in June 2013 and the vegetation sampling campaign commenced in December 2013. Further details of the experimental design can be found in Power et al. (2016) [26].

**Fig. 5:**
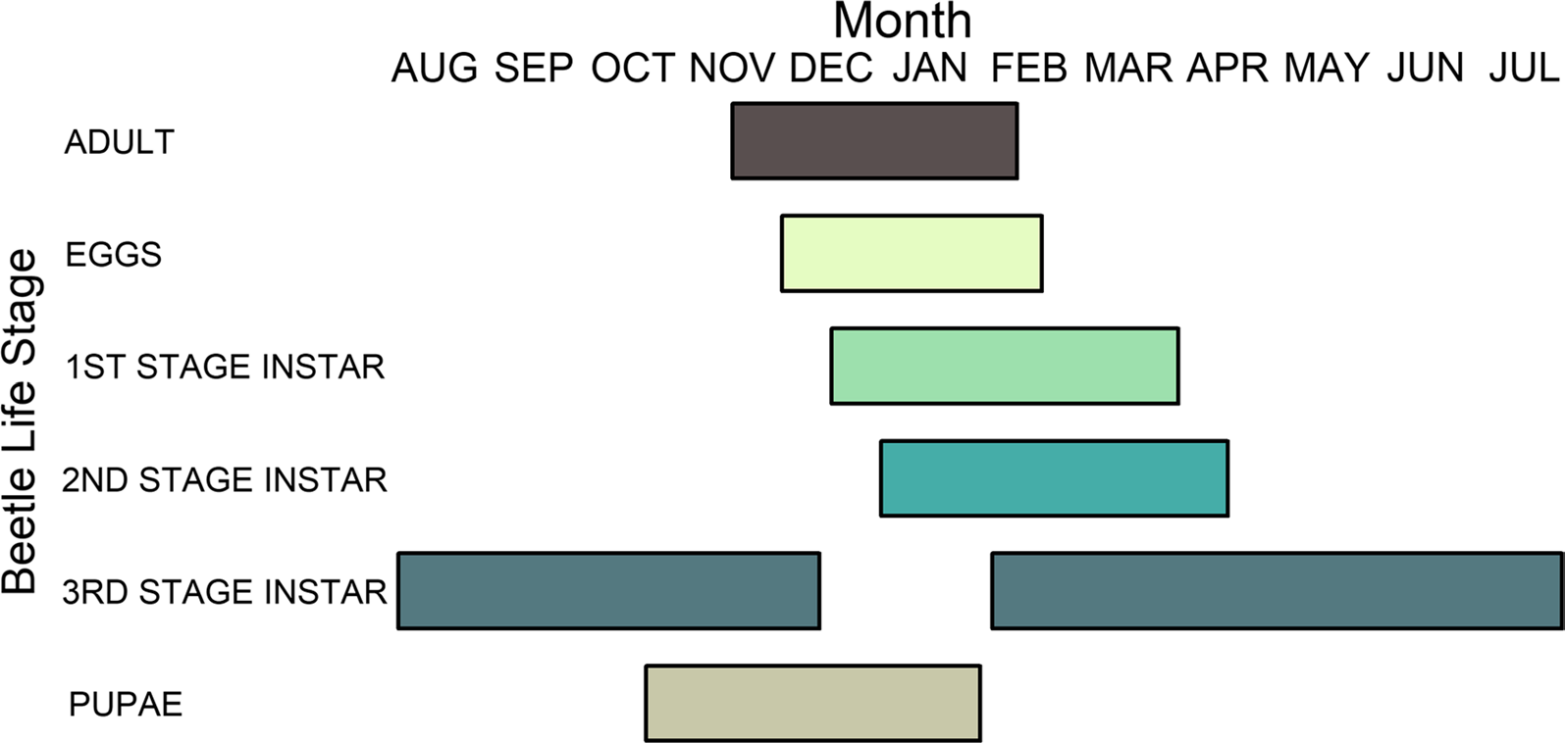
Seasonal occurrence of the life stages for a common scarab pest species in SE Australia (*Cyclocephala signaticolis*). Color of bars indicate each scarab life stage and when it typically occurs. Data were obtained from Frew et al. (2016) [93]

Root-feeding scarabs are a known pest in this area of Australia and are prone to outbreaks under certain climatic conditions [82, 83]. While little research has been done on belowground beetle outbreaks and climate change, outbreaks of phytophagous beetles have been linked to changes in climate [84]. We selected a few of these local scarabs (*Sericesthis* spp., *Heteronychus arator*, *Cyclocephala signaticollis*, and *Othnonius batesi*) (see Fig. 5 for a scarab life cycle example), which can be found in large numbers in some years, to simulate an outbreak in response to climatic changes. Root herbivore treatments were applied using a supplementation approach, by allowing adult scarab beetles to oviposit within plots to produce root-feeding offspring [85]. Beetles were collected by light trapping in the local area and applied (December 2013 and 2014 and January 2014 and 2015) to half of the 36 plots (RH_+_ plots) with the remaining plots being non-supplemented (RH_0_ plots); no pre-treatment beetle measurements were taken on the plots due to the destructive nature of such sampling. Adult beetles were enclosed on RH_+_ plots using mosquito netting stretched along a pegged-in bamboo frame (1 m × 1 m), and allowed to oviposit for 3–5 days. The RH_+_ plots received an unevenly mixed population (c. 27 g fresh weight; the average catch per night) of adult scarab beetles of the species mentioned above. Netting was held away from the vegetation by fixing the top portion to the underside of the shelter roof. All RH_+_ plots were inoculated twice per warm season. Empty nets were also placed on RH_0_ plots to control for effects of the netting itself on vegetation.

### Plant sampling

Surveys of plant cover were carried out in August 2013, February 2014, September 2014, February 2015 and August 2015. These involved placing a 1 m^2^ quadrat, divided into 25 cells, on top of vegetation in the centre of the plot. Grasses, legumes and forbs were identified to species, and scored on a presence/absence basis for each cell of the quadrat to derive a cover frequency value for each plot (i.e. cell count of each species added together), which was then averaged per season (cool: August 2013, September 2014, August 2015 | warm: February 2014, February 2015). Biomass harvests were taken at two points in the year, once at the end of the cool season (September–October 2013 & 2014) and once at the end of the warm season (March–April 2014 & 2015). Plot-level biomass removal twice-yearly reflects grazing management practice in the local area and prevents accumulation of a very large amount of standing dead thatch that inhibits seedling establishment and growth of all except the most competitive species. Plant regrowth following twice-yearly biomass removal was vigorous and rapid, and the study is carried out in the context of this simulated grazing removal. Vegetation in the central 1 m^2^ was cut to ground level and a 20% (by fresh weight) subsample of all material was sorted to dead and live, with the live material further sorted to species level. Plant material was then dried for 72 h at 70 °C and weighed. The most frequent species (in terms of both biomass and cover) are listed in Table 1. Because cover and biomass were sampled at different time periods, some species may not have data for both cover and biomass.

**Table 1:** List of the most abundant plant species/genera and their total biomass (g m^−2^)/cover (%) estimates for all plots during the course of the experiment

Species	C_3_/C_4_	Functional group	Life cycle	Warm season cover	Warm season biomass	Cool season cover	Cool season biomass
*Avena* spp.	C_3_	grass	Annual	0	0	0.25	0
*Bromus catharticus*	C_3_	grass	Annual	0	0	0.67	46.3
*Digitaria* sp.	C_3_	grass	Annual	1.13	481.2	0.28	32.2
*Gamochaeta* spp.	C_3_	forb	Annual	0.17	0	0.44	0
*Modiola caroliniana*	C_3_	forb	Annual	0	0.12	0.28	0
*Ornithopus compressus*	C_3_	legume	Annual	0.46	0.18	2.07	88.1
*Oxalis corniculata*	C_3_	legume	Annual	0.79	3.32	3.1	0.59
*Sonchus oleraceus*	C_3_	forb	Annual	0	0	0.8	8.61
*Vicia sativa*	C_3_	legume	Annual	0	0.11	9.13	153.8
*Anagallis arvensis*	C_3_	forb	Annual/Biennial	0	0	0.27	0.98
*Plantago lanceolata*	C_3_	forb	Annual/Biennial	2.06	40.7	4.62	400.4
*Senecio madagascariensis*	C_3_	forb	Annual/Biennial	0.66	1.25	2.67	207.5
*Axonopus fissifolius*	C_4_	grass	Perennial	9.14	345.4	6.63	225.7
*Bothriochloa macra*	C_4_	grass	Perennial	4.12	824.5	2.02	4.05
*Cymbopogon refractus*	C_4_	grass	Perennial	9.45	1641.7	5.01	209.9
*Cynodon dactylon*	C_4_	grass	Perennial	13.4	1157.8	7.81	1149.6
*Eragrostis curvula*	C_4_	grass	Perennial	2.14	1989.7	4.32	2113.3
*Hypochaeris radicata*	C_3_	forb	Perennial	4.25	25.6	8.34	142
*Lolium perenne*	C_3_	grass	Perennial	0	0	8.56	813.2
*Lotus corniculatus*	C_3_	legume	Perennial	0.57	0.53	2.25	4.97
*Microlaena stipoides*	C_3_	grass	Perennial	13.2	516.6	11.72	993.3
*Paspalum* spp.	C_4_	grass	Perennial	20.9	3548.7	13.18	1077.8
*Setaria* *parviflora*	C_4_	grass	Perennial	10.2	1520.1	3.46	92.5
*Verbena* spp.	C_3_	forb	Perennial	0.39	27.2	0.47	99.4
Other (unidentified)	various	various	Annual/Biennial/Perennial	6.98	218.3	1.64	63.2

### Root cores & soil invertebrate extraction

In December 2013, mesh-free ingrowth cores (6 cm diameter, 10 cm depth) were established (3 per plot) [86] in order to monitor treatment effects on root productivity. Soil removed during the initial coring provided a measure of root standing crop. A 5 cm diameter piece of orange PVC was then placed to a depth of 2 cm to mark the location of in-growth cores. These were backfilled with sieved local soil and packed to similar density as surrounding soil. Ingrowth cores were left for 6 months before being re-sampled, sieved (2 mm mesh) and hand-sorted. Samples were then dried at 70 °C for 48 h and weighed. Ingrowth cores were re-sampled at 6 month intervals from December 2013 to July 2015.

Background levels of root herbivore activity were assumed not to vary systematically across treatments at the beginning of the experiment; this was not measured directly due to the destructive nature of such sampling. However, in order to determine the effectiveness of scarab beetle inoculations, belowground invertebrates were sampled in September 2015 in a destructive excavation of small sub-plots within each plot (Additional file 1: Fig. A2). Two 25 cm × 10 cm × 20 cm (length × width × depth) holes were excavated on the east and west edge of each plot, just outside of the central 1 m^2^. Excavated soil was sieved to 2 mm and recovered invertebrates were stored in ethanol and placed in a freezer prior to identification in the laboratory under a dissection microscope (SZ51, Olympus, Japan). While this did not provide an accurate estimate of larval survival or root damage, it allowed verification of the extent to which inoculations increased root herbivore abundance in the treated plots.

### Statistics

All statistical analyses were performed using R, version 3.3.2 [87]. Volumetric soil water content (SWC %) was aggregated by month (December 2 2013–December 2 2015) and logit transformed. Sporadic sensor and datalogging errors meant that the data were slightly unbalanced and therefore were analysed with a repeated measures linear mixed effects model (fixed: season*rain-treatment + rainfall [collected by sensor on site as covariate], random: plot) with type 3 sums of squares; models were compared via AIC and a pairwise post hoc analyses were performed with a Benjamini–Hochberg correction on linear mixed effects models [88, 89]. Variation in SWC across seasons and treatments was calculated with a CV [90]. Root productivity data were normalised by scaling to values between 0 and 1 for each sampling event (i.e. subtracting the minimum value from each value and then dividing by the range). Analyses of treatment effects were carried out on scaled, logit transformed data. Seasons were designated as either warm (collected November–April) or cool (collected May–October). Because there were an unequal number of sample seasons with root-herbivore added (two warm to one cool), warm season biomass data were averaged from both sampling periods, where appropriate. Data were checked for normality, sphericity and homogeneity of variance. Transformed root mass data were analysed with two models: (1) a linear model with only pre-root herbivore-added standing crop as a response to evaluate the existing state of roots mass in plots (fixed: rain-treatment*root herbivore-addition) and (2) a repeated measures linear mixed effects model, without the standing crop included in the response (fixed: season*rain-treatment*herbivore-addition + standing crop per plot [as a covariate], random: plot) with type 3 sums of squares; models were compared via AIC and a post hoc analysis with a Benjamini–Hochberg correction was performed pairwise on linear mixed effects models, by rain treatment. This model was created to test for the effect of rain treatments, root herbivore addition, and to see if/how this relationship varied by season.

Rainfall treatment, root herbivore addition and their interaction were evaluated for effects on plant community biomass, cover, diversity (Shannon’s H) and evenness. Total and dead plant biomass data were not normally distributed and were therefore analysed by permutation anova (adonis()) using contrasts that were not order-dependent, 999 permutations and with a ‘Euclidean’ distance method [91]. Species diversity (*Shannon–Wiener index*) and evenness (*Shannon’s equitability*) were calculated using the *vegan* package [92] and transformed to conform to normality. All post hoc analyses were performed pairwise, manually for all permutation anovas. Additionally, because the root herbivore treatment was not introduced until after the first cool season cover survey (August 2013) and biomass harvest (October 2013), analyses of root herbivore treatment effects were restricted to data collected after these dates; a separate analysis was run on pre-treatment data for these surveys. Species were designated as C_3_ or C_4_ based on their photosynthetic pathway and C_3_:C_4_ ratio was calculated for each plot using both biomass and cover data. All univariate plant data were evaluated with adonis (*vegan*; [92]) within R, using a ‘Euclidean’ distance matrix.

## Supplementary Information

**Additional file 1: Table A1.** Plant responses to rainfall treatments, herbivore addition, and season: cover (frequency per m^2^) estimates for all plots during February 2014 through August 2015 of the experiment. **Table A2.** Plant responses to rainfall treatments and season: cover (frequency per m^2^) estimates for all plots during February 2014 through August 2015 of the experiment. **Table A3.** Plant responses to season: cover (frequency per m^2^) estimates for all plots during February 2014 through August 2015 of the experiment. **Table A4.** Cover means over time. **Table A5.** Plant responses to rainfall treatments, herbivore addition, and season: dry live + dead mass (g/m^2^) estimates for all plots during October 2014 through April 2015 of the experiment. **Table A6.** Plant responses to rainfall treatments and season: dry live + dead mass (g/m^2^) estimates for all plots during October 2014 through April 2015 of the experiment. **Table A7.** Plant responses to season: dry live + dead mass (g/m^2^) estimates for all plots during October 2014 through April 2015 of the experiment. **Table A8.** Plant responses to season: dry live + dead mass (g/m^2^) estimates for all plots over time. **Table A9.** Plant responses to rainfall treatments, herbivore addition, and season: dry live mass (g/m^2^) estimates for all plots during October 2014 through April 2015 of the experiment. **Table A10.** Plant responses to rainfall treatments and season: dry live mass (g/m^2^) estimates for all plots during October 2014 through April 2015 of the experiment. **Table A11.** Plant responses to season: dry live mass (g/m^2^) estimates for all plots during October 2014 through April 2015 of the experiment. **Table A12.** Plant responses to scarab addition: dry live mass (g/m^2^) estimates during October 2013 (pre-scarab) through April 2015 of the experiment. **Table A13.** Plant responses to season: dry live (g/m^2^) estimates for all plots over time. **Table A14.** Plant responses to rainfall treatments, herbivore addition, and season: dry dead mass (g/m^2^) estimates for all plots during October 2014 through April 2015 of the experiment. **Table A15.** Plant responses to rainfall treatments and season: dry dead mass (g/m^2^) estimates for all plots during October 2014 through April 2015 of the experiment. **Table A16.** Plant responses to season: dry dead mass (g/m^2^) estimates for all plots during October 2014 through April 2015 of the experiment. **Table A17.** Plant responses to season: dry dead mass (g/m^2^) estimates for all plots over time. **Table A18.** Dry biomass from root-ingrowth core samples over the course of the experiment. **Table A19.** Dry biomass from root-ingrowth core samples over the course of the experiment normalized (due to diminishing returns: per season treatments/per season amb/RH_0_). **Table A20.** Biomass-based C_3_/C_4_ ratios. **Table A21.** Cover-based C_3_/C_4_ ratios. At cool season 2013, the herbivore treatment was not yet added. **Table A22.** Biomass-based diversity (Shannon–Wiener *H*). **Table A23.** Biomass-based evenness. **Table A24.** Cover-based diversity (Shannon–Wiener *H*). **Table A25.** Cover-based evenness. **Table A26.** Soil moisture (%) repeated measures linear mixed effects model (fixed: season*rain-treatment + rainfall anova) table. **Table A27.** Plant cover permanova analysis. **Table A28.** Plant cover permanova analysis pre-scarab addition. **Table A29.** Plant total (live + dead) biomass permanova analysis. **Table A30.** Plant total (live + dead) biomass permanova analysis pre-scarab addition. **Table A31.** Plant live biomass permanova analysis. **Table A32.** Plant live biomass permanova analysis pre-scarab addition. **Table A33.** Plant dead biomass permanova analysis. **Table A34.** Plant dead biomass permanova analysis. **Table A35.** Root biomass repeated measures ANOVA (Type III). **Table A36.** Root biomass repeated measures ANOVA (Type III) pre-scarab addition. **Table A37.** Cover C3:C4 ratio permanova analysis. **Table A38.** Cover C3:C4 ratio permanova analysis pre-scarab addition. **Table A39.** Biomass C3:C4 ratio permanova analysis. **Table A40.** Biomass C3:C4 ratio permanova analysis pre-scarab addition. **Table A41. Plant** diversity (Shannon’s H), based on cover, permanova analysis. **Table A42.** Plant diversity (Shannon’s H), based on cover, permanova analysis pre-scarab addition. **Table A43.** Plant diversity (Shannon’s H), based on biomass, permanova analysis. **Table A44.** Plant diversity (Shannon’s H), based on biomass, permanova analysis pre-scarab addition. **Table A45.** Plant evenness, based on cover, permanova analysis. **Table A46.** Plant evenness, based on cover, permanova analysis pre-scarab addition. **Table A47.** Plant evenness, based on biomass, permanova analysis. **Table A48.** Plant evenness, based on biomass, permanova analysis pre-scarab addition. **Table A49.** Scarab extraction zero-inflated count model, with least ratio comparison with and without RH in the model. **Table A50.** Per plot percentage of live biomass for the top 4 species found in all seasons. **Figure A1.** A) Soil moisture (%SWC) and simulated rain (mm) 2013-12-02 to 2015-12-02; dashed line represents mean, B) Soil moisture (%SWC) and simulated rain (mm) over the warmest portion of 1 year 2014-09-01 to 2015-01-28; dashed line represents mean. **Figure A2.** Scaled scarab larva numbers within sub plots (Two 25 cm × 10 cm × 20 cm holes) within each grassland plot. **Figure A3.**  Herbivore netting used to hold beetles within treatment plots; sides were held down with pegs. Tents were removed during peak daylight hours.** Figure A4**. Scaled and normalized dry root mass obtained from root-ingrowth cores. RA – Reduced Amount rainfall, RF – Reduced Frequency rainfall, RH_0_ – No scarabs added, RH_+_ – Scarabs added.

## Data Availability

All data need to evaluate the conclusions in the paper are present in the paper and/or Additional file 1. The R code and raw data used and/or analysed during the current study are available from the corresponding author on reasonable request.

## Abbreviations

C_3_: Plants that predominantly use the C_3_ photosynthetic pathway
C_4_: Plants that predominantly use the C_4_ photosynthetic pathway
SWC: Soil water content
Amb: Ambient rainfall treatment
RA: Reduced amount rainfall treatment
RF: Reduced frequency rainfall treatment
CV: Coefficient of variation (stdev/mean)
RH_0_: Root herbivore background treatment
RH_+_: Root herbivore added treatment
DRI-Grass: Drought and Root herbivore Impacts on Grassland
C:N: Carbon to nitrogen ratio
PVC: Polyvinyl chloride

## References

[1] Wang W, Fang J (2009). Soil respiration and human effects on global grasslands. Glob Planet Change.

[2] Ojima DS, Parton WJ, Schimel DS, Scurlock JMO, Kittel TGF. Modeling the effects of climatic and CO_2_ changes on grassland storage of soil C. In: Wisniewski J, Sampson RN, editors. Terrestrial biospheric carbon fluxes quantification of sinks and sources of CO_2_. Dordrecht: Springer Netherlands; 1993. p. 643–57. 10.1007/978-94-011-1982-5_43.

[3] Lenhart PA, Eubanks MD, Behmer ST (2015). Water stress in grasslands: dynamic responses of plants and insect herbivores. Oikos.

[4] Knapp AK, Fay PA, Blair JM, Collins SL, Smith MD, Carlisle JD (2002). Rainfall variability, carbon cycling, and plant species diversity in a mesic grassland. Science.

[5] Huxman TE, Snyder KA, Tissue D, Leffler AJ, Ogle K, Pockman WT (2004). Precipitation pulses and carbon fluxes in semiarid and arid ecosystems. Oecologia.

[6] Fischer E, Beyerle U, Knutti R (2013). Robust spatially aggregated projections of climate extremes. Nat Clim Change.

[7] Kreyling J, Beier C (2013). Complexity in climate change manipulation experiments. Bioscience.

[8] Taylor SH, Ripley BS, Martin T, De-Wet LA, Woodward FI, Osborne CP (2014). Physiological advantages of C_4_ grasses in the field: a comparative experiment demonstrating the importance of drought. Glob Change Biol.

[9] Adler PB, Levine JM (2007). Contrasting relationships between precipitation and species richness in space and time. Oikos.

[10] Robertson TR, Zak JC, Tissue DT (2010). Precipitation magnitude and timing differentially affect species richness and plant density in the sotol grassland of the Chihuahuan Desert. Oecologia.

[11] Lee MA, Manning P, Walker CS, Power SA (2014). Plant and arthropod community sensitivity to rainfall manipulation but not nitrogen enrichment in a successional grassland ecosystem. Oecologia.

[12] Barnett KL, Facey SL (2016). Grasslands, invertebrates, and precipitation: a review of the effects of climate change. Front Plant Sci.

[13] Pritchard PM. Reproductive capacity of grape root borer, *Vitacea polistiformis* (Harris), and implications for pheromone based management. Doctor of Philosophy. North Carolina State University; 2004.

[14] Jactel H, Koricheva J, Castagneyrol B (2019). Responses of forest insect pests to climate change: not so simple. Curr Opin Insect Sci.

[15] Johnson SN, Erb M, Hartley SE (2016). Roots under attack: contrasting plant responses to below-and aboveground insect herbivory. New Phytol.

[16] Johnson SN, Benefer CM, Frew A, Griffiths BS, Hartley SE, Karley AJ (2016). New frontiers in belowground ecology for plant protection from root-feeding insects. Appl Soil Ecol.

[17] Seastedt TR, Murray PJ, Johnson SN, Murray PJ (2008). Root herbivory in grassland ecosystems. Root feeders: an ecosystem perspective.

[18] Zvereva EL, Kozlov MV (2012). Sources of variation in plant responses to belowground insect herbivory: a meta-analysis. Oecologia.

[19] Staley JT, Mortimer SR, Morecroft MD, Brown VK, Masters GJ (2007). Summer drought alters plant-mediated competition between foliar- and root-feeding insects. Glob Change Biol.

[20] Johnson SN, Staley JT, McLeod FAL, Hartley SE (2011). Plant-mediated effects of soil invertebrates and summer drought on above-ground multitrophic interactions. J Ecol.

[21] Schallhart N, Tusch MJ, Wallinger C, Staudacher K, Traugott M (2012). Effects of plant identity and diversity on the dietary choice of a soil-living insect herbivore. Ecology.

[22] Caswell H, Reed F, Stephenson SN, Werner PA (1973). Photosynthetic pathways and selective herbivory: a hypothesis. Am Nat.

[23] Johnson SN, Lopaticki G, Hartley SE (2014). Elevated atmospheric CO_2_ triggers compensatory feeding by root herbivores on a C_3_ but not a C_4_ grass. PLoS One.

[24] Gerard PJ, Popay AJ, Johnson SN, Jones TH (2017). Climate change effects on biological control in grasslands. Global climate change and terrestrial invertebrates.

[25] Fry EL, Manning P, Allen DGP, Hurst A, Everwand G, Rimmler M, et al. Plant functional group composition modifies the effects of precipitation change on grassland ecosystem function. PLoS ONE. 2013;8:e57027. 10.1371/journal.pone.0057027 PMC3577764 23437300

[26] Power SA, Barnett KL, Ochoa Hueso R, Facey SL, Gibson-Forty EVJ, Nielsen UN (2016). DRI-Grass: a new experimental platform for addressing grassland ecosystem responses to future precipitation scenarios in south-east Australia. Front Plant Sci.

[27] CSIRO, Australian Bureau of Meteorology. State of the Climate 2012. Canberra: CSIRO; 2012.

[28] Australian Bureau of Meteorology. Australian climate variability & change—Trend maps (Rainfall—Total). Australian Bureau of Meteorology. 2013. http://www.bom.gov.au/climate/change/. Accessed 13 Mar 2016.

[29] Deléglise C, Meisser M, Mosimann E, Spiegelberger T, Signarbieux C, Jeangros B (2015). Drought-induced shifts in plants traits, yields and nutritive value under realistic grazing and mowing managements in a mountain grassland. Agric Ecosyst Environ.

[30] Fay PA, Carlisle JD, Knapp AK, Blair JM, Collins SL (2003). Productivity responses to altered rainfall patterns in a C_4_-dominated grassland. Oecologia.

[31] Harper CW, Blair JM, Fay PA, Knapp AK, Carlisle JD (2005). Increased rainfall variability and reduced rainfall amount decreases soil CO_2_ flux in a grassland ecosystem. Glob Change Biol.

[32] Knapp AK, Beier C, Briske DD, Classen AT, Luo Y, Smith MD (2008). Consequences of more extreme precipitation regimes for terrestrial ecosystems. Bioscience.

[33] Röttgermann M, Steinlein T, Beyschlag W, Dietz H (2000). Linear relationships between aboveground biomass and plant cover in low open herbaceous vegetation. J Veg Sci.

[34] Luzuriaga AL, Escudero A, Loidi J (2002). Above-ground biomass distribution among species during early old-field succession. J Veg Sci.

[35] Brown G (2003). Species richness, diversity and biomass production of desert annuals in an ungrazed *Rhanterium epapposum* community over three growth seasons in Kuwait. Plant Ecol.

[36] Garnier E (1991). Above and below-ground resource capture in herbaceous plants: relationships with growth and biomass allocation. Trends Ecol Evol.

[37] Holmes T, Rice K (1996). Patterns of growth and soil-water utilization in some exotic annuals and native perennial bunchgrasses of California. Ann Bot.

[38] Dyer AR, Rice KJ (1999). Effects of competition on resource availability and growth of a California bunchgrass. Ecology.

[39] Corbin JD, D’Antonio CM (2004). Competition between native perennial and exotic annual grasses: implications for an historical invasion. Ecology.

[40] Ledgard SF, Steele KW (1992). Biological nitrogen fixation in mixed legume/grass pastures. Plant Soil.

[41] Serraj R, Sinclair TR, Purcell LC (1999). Symbiotic N_2_ fixation response to drought. J Exp Bot.

[42] Clarke PJ, Latz PK, Albrecht DE (2005). Long-term changes in semi-arid vegetation: invasion of an exotic perennial grass has larger effects than rainfall variability. J Veg Sci.

[43] Cook GD, Dias L (2006). It was no accident: deliberate plant introductions by Australian government agencies during the 20th century. Aust J Bot.

[44] Gallagher RV, Beaumont LJ, Hughes L, Leishman MR (2010). Evidence for climatic niche and biome shifts between native and novel ranges in plant species introduced to Australia. J Ecol.

[45] Farooq M, Wahid A, Kobayashi N, Fujita D, Basra SMA (2009). Plant drought stress: effects, mechanisms and management. Agron Sustain Agric.

[46] Heckathorn SA, DeLucia EH (1994). Drought-induced nitrogen retranslocation in perennial C_4_ grasses of tallgrass prairie. Ecology.

[47] Parton WJ, Scurlock JMO, Ojima DS, Gilmanov TG, Scholes RJ, Schimel DS (1993). Observations and modeling of biomass and soil organic matter dynamics for the grassland biome worldwide. Glob Biogeochem Cycles.

[48] Fry EL, Manning P, Power SA (2014). Ecosystem functions are resistant to extreme changes to rainfall regimes in a mesotrophic grassland. Plant Soil.

[49] Barnes PW, Harrison AT (1982). Species distribution and community organization in a Nebraska sandhills mixed prairie as influenced by plant/soil-water relationships. Oecologia.

[50] Pearcy RW, Ehleringer J (1984). Comparative ecophysiology of C_3_ and C_4_ plants. Plant Cell Environ.

[51] Huang B, Duncan RR, Carrow RN (1997). Drought-resistance mechanisms of seven warm-season turfgrasses under surface soil drying: II. Root aspects. Crop Sci.

[52] Gibson-Forty EVJ, Barnett KL, Tissue DT, Power SA (2016). Reducing rainfall amount has a greater negative effect on the productivity of grassland plant species than reducing rainfall frequency. Funct Plant Biol.

[53] Nie ZN, Miller S, Moore GA, Hackney BF, Boschma SP, Reed KFM, et al. Field evaluation of perennial grasses and herbs in southern Australia. 2. Persistence, root characteristics and summer activity. Aust J Exp Agric. 2008;48:424–35.

[54] Pfisterer AB, Schmid B (2002). Diversity-dependent production can decrease the stability of ecosystem functioning. Nature.

[55] Van Peer L, Nijs I, Reheul D, De Cauwer B (2004). Species richness and susceptibility to heat and drought extremes in synthesized grassland ecosystems: compositional vs physiological effects. Funct Ecol.

[56] Wang Y, Zhai X, Sun H, Chang H, Rong L (2019). Performance of different sampling methods in estimating species richness in semiarid grassland. Ecol Eng.

[57] Catchpole WR, Wheeler CJ (1992). Estimating plant biomass: a review of techniques. Aust J Ecol.

[58] Ryser P, Langenauer R, Gigon A (1995). Species richness and vegetation structure in a limestone grassland after 15 years management with six biomass removal regimes. Folia Geobot.

[59] Weltzin JF, Keller JK, Bridgham SD, Pastor J, Allen PB, Chen J (2005). Litter controls plant community composition in a northern fen. Oikos.

[60] Heisler-White JL, Blair JM, Kelly EF, Harmoney K, Knapp AK (2009). Contingent productivity responses to more extreme rainfall regimes across a grassland biome. Glob Change Biol.

[61] Sonnemann I, Hempel S, Beutel M, Hanauer N, Reidinger S, Wurst S (2013). The root herbivore history of the soil affects the productivity of a grassland plant community and determines plant response to new root herbivore attack. PLoS ONE.

[62] Birhane E, Sterck FJ, Fetene M, Bongers F, Kuyper TW (2012). Arbuscular mycorrhizal fungi enhance photosynthesis, water use efficiency, and growth of frankincense seedlings under pulsed water availability conditions. Oecologia.

[63] Kostenko O, van de Voorde TFJ, Mulder PPJ, van der Putten WH, Martijn BT (2012). Legacy effects of aboveground–belowground interactions. Ecol Lett.

[64] Huber M, Bont Z, Fricke J, Brillatz T, Aziz Z, Gershenzon J (2016). A below-ground herbivore shapes root defensive chemistry in natural plant populations. Proc R Soc B.

[65] Buckley YM, Rees M, Sheppard AW, Smyth MJ (2005). Stable coexistence of an invasive plant and biocontrol agent: a parameterized coupled plant–herbivore model. J Appl Ecol.

[66] King PD, Mercer CF, Meekings JS (1981). Ecology of black beetle, *Heteronychus arator* (Coleoptera: Scarabaeidae)-population modelling. N Z J Agric Res.

[67] Ridsdill-Smith TJ (1977). Effects of root-feeding by scarabaeid larvae on growth of perennial ryegrass plants. J Appl Ecol.

[68] Gerber E, Hinz HL, Blossey B (2007). Impact of the belowground herbivore and potential biological control agent, *Ceutorhynchus scrobicollis*, on *Alliaria petiolata* performance. Biol Control.

[69] Barnett KL, Johnson SN, Power SA (2018). Drought negates growth stimulation due to root herbivory in pasture grasses. Oecologia.

[70] Gerard PJ, Hackell DL, Bell NL (2007). Impact of clover root weevil *Sitona lepidus* (Coleoptera: Curculionidae) larvae on herbage yield and species composition in a ryegrass-white clover sward. N Z J Agric Res.

[71] Barbehenn RV (1992). Digestion of uncrushed leaf tissues by leaf-snipping larval lepidoptera. Oecologia.

[72] Barbehenn RV, Chen Z, Karowe DN, Spickard A (2004). C_3_ grasses have higher nutritional quality than C_4_ grasses under ambient and elevated atmospheric CO_2_. Glob Change Biol.

[73] Stevens GNG, Pierson DDR, Nguyen K, Jones RRH (2007). Differences between resource patches modify root herbivore effects on plants. Plant Soil.

[74] Epstein HE, Lauenroth WK, Burke IC, Coffin DP. Productivity patterns of C_3_ and C_4_ functional types in the U.S. Great Plains. Ecology. 1997;78:722–31.

[75] Gerard PJ, Barringer JRF, Charles JG, Fowler SV, Kean JM, Phillips CB (2013). Potential effects of climate change on biological control systems: case studies from New Zealand. BioControl.

[76] Torode MD, Barnett KL, Facey SL, Nielsen UN, Power SA, Johnson SN (2016). Altered precipitation impacts on above-and below-ground grassland invertebrates: summer drought leads to outbreaks in spring. Front Plant Sci.

[77] Isbell R. The Australian Soil Classification. Handbook. Melbourne, VIC: CSIRO; 2002. http://www.publish.csiro.au/pid/3529.htm.

[78] Barton CVM, Ellsworth DS, Medlyn BE, Duursma RA, Tissue DT, Adams MA (2010). Whole-tree chambers for elevated atmospheric CO_2_ experimentation and tree scale flux measurements in south-eastern Australia: the Hawkesbury Forest Experiment. Agric For Meteorol.

[79] Peel MC, Finlayson BL, McMahon TA (2007). Updated world map of the Köppen-Geiger climate classification. Hydrol Earth Syst Sci Discuss.

[80] Australian Bureau of Meteorology. Australian climate variability & change. Australian Bureau of Meteorology. 2016. http://www.bom.gov.au/climate/change/. Accessed 20 Mar 2016.

[81] IPCC (2013). Climate change 2013: the physical science basis.

[82] Matthiessen JN, Ridsdill-Smith TJ (1991). Populations of African black beetle, *Heteronychus arator* (Coleoptera: Scarabaeidae) in a Mediterranean climate region of Australia. Bull Entomol Res.

[83] Karpyn Esqueda M, Yen AL, Rochfort S, Guthridge KM, Powell KS, Edwards J (2017). A review of perennial ryegrass endophytes and their potential use in the management of African Black Beetle in perennial grazing systems in Australia. Front Plant Sci.

[84] Rojas MR, Locatelli B, Billings R (2010). Climate change and outbreaks of Southern Pine Beetle *Dendroctonus frontalis* in Honduras. For Syst.

[85] Johnson SN, Crotty FV, Ryalls JMW, Murray PJ, Ohgushi T, Wurst S, Johnson SN (2018). Belowground experimental approaches for exploring aboveground-belowground patterns. Aboveground-belowground community ecology.

[86] Raich JW, Riley RH, Vitousek PM (1994). Use of root-ingrowth cores to assess nutrient limitations in forest ecosystems. Can J For Res.

[87] R Core Team. R: a language and environment for statistical computing. Vienna, Austria: R Foundation for Statistical Computing; 2016. https://www.R-project.org/.

[88] Benjamini Y, Hochberg Y (1995). Controlling the false discovery rate: a practical and powerful approach to multiple testing. J R Stat Soc Ser B.

[89] Hothorn T, Bretz F, Westfall P (2008). Simultaneous inference in general parametric models. Biometr J.

[90] Le Houerou HN, Bingham RL, Skerbek W (1988). Relationship between the variability of primary production and the variability of annual precipitation in world arid lands. J Arid Environ.

[91] McArdle BH, Anderson MJ (2001). Fitting multivariate models to community data: a comment on distance-based redundancy analysis. Ecology.

[92] Oksanen J, Blanchet FG, Kindt R, Legendre P, Minchin PR, O’Hara RB, et al. vegan: Community Ecology Package. vegan: Community Ecology Package. 2016. https://CRAN.R-project.org/package=vegan.

[93] Frew A, Barnett K, Riegler M, Nielsen UN, Johnson SN (2016). Belowground ecology of scarabs feeding on grass roots: current knowledge and future directions for management in Australasia. Front Plant Sci.

