# Supplementary material for: Altered precipitation and root herbivory affect the productivity and composition of a mesic grassland
Source: BMC Ecol Evol. 2021 Jul 15;21:145. doi: 10.1186/s12862-021-01871-0 (PMC8283849; doi:10.1186/s12862-021-01871-0)

Table A1. Plant responses to rainfall treatments, herbivore addition, and season: cover (frequency per m^2^) estimates for all plots during February 2014 through August 2015 of the experiment. Amb – Ambient rainfall, RA – Reduced Amount rainfall, RF – Reduced Frequency rainfall, RH_0_ – No scarabs added, RH_+_ - Scarabs added.

| **Plant Cover (Feb 2014-Aug 2015)** | | | | |
| --- | --- | --- | --- | --- |
| **Season** | **Rainfall** | **RH** | **Cover Freq.** | **SE** |
| Warm | Amb | RH_0_ | 98.8 | 5.6 |
| Warm | Amb | RH_+_ | 95.8 | 5.1 |
| Warm | RA | RH_0_ | 83.2 | 7.3 |
| Warm | RA | RH_+_ | 79.4 | 8.4 |
| Warm | RF | RH_0_ | 83.8 | 6.7 |
| Warm | RF | RH_+_ | 85.4 | 6.8 |
| Cool | Amb | RH_0_ | 132.3 | 6.7 |
| Cool | Amb | RH_+_ | 133.9 | 7.8 |
| Cool | RA | RH_0_ | 100.5 | 3.9 |
| Cool | RA | RH_+_ | 106.7 | 4.1 |
| Cool | RF | RH_0_ | 114.2 | 5.7 |
| Cool | RF | RH_+_ | 115.8 | 5.9 |

Table A2. Plant responses to rainfall treatments and season: cover (frequency per m^2^) estimates for all plots during February 2014 through August 2015 of the experiment. Amb – Ambient rainfall, RA – Reduced Amount rainfall, RF – Reduced Frequency rainfall, RH_0_ – No scarabs added, RH_+_ - Scarabs added.

| **Plant Cover (Feb 2014-Aug 2015)** | | | |
| --- | --- | --- | --- |
| **Season** | **Rainfall** | **Cover Freq.** | **SE** |
| Warm | Amb | 97.2 | 3.6 |
| Warm | RA | 81.3 | 5.3 |
| Warm | RF | 84.6 | 4.6 |
| Cool | Amb | 133.1 | 4.9 |
| Cool | RA | 103.6 | 2.8 |
| Cool | RF | 115.0 | 3.9 |

Table A3. Plant responses to season: cover (frequency per m^2^) estimates for all plots during February 2014 through August 2015 of the experiment. Amb – Ambient rainfall, RA – Reduced Amount rainfall, RF – Reduced Frequency rainfall, RH_0_ – No scarabs added, RH_+_ - Scarabs added.

| **Plant Cover (Feb 2014-Aug 2015)** | | |
| --- | --- | --- |
| **Season** | **Cover Freq.** | **SE** |
| Warm | 87.7 | 2.3 |
| Cool | 117.2 | 2.9 |

**Table A4. Cover means over time. At cool season 2013, the herbivore treatment was not yet added. Amb – Ambient rainfall, RA – Reduced Amount rainfall, RF – Reduced Frequency rainfall, RH0 – No scarabs added, RH+ - Scarabs added.**

| **Season** | **Rainfall** | **RH** | **Cover Freq.** | **SE** |
| --- | --- | --- | --- | --- |
| Cool 2013 | Amb | RH0 | 155.3 | 8.4 |
| Cool 2013 | Amb | RH+ | 159.8 | 6.3 |
| Cool 2013 | RA | RH0 | 148.2 | 11.1 |
| Cool 2013 | RA | RH+ | 150.5 | 8.3 |
| Cool 2013 | RF | RH0 | 156.5 | 8.0 |
| Cool 2013 | RF | RH+ | 130.2 | 8.9 |
| Warm 2014 | Amb | RH0 | 91.5 | 7.7 |
| Warm 2014 | Amb | RH+ | 96.0 | 7.3 |
| Warm 2014 | RA | RH0 | 77.2 | 9.6 |
| Warm 2014 | RA | RH+ | 71.7 | 7.7 |
| Warm 2014 | RF | RH0 | 78.7 | 7.9 |
| Warm 2014 | RF | RH+ | 79.7 | 7.3 |
| Cool 2014 | Amb | RH0 | 125.0 | 9.8 |
| Cool 2014 | Amb | RH+ | 130.7 | 12.5 |
| Cool 2014 | RA | RH0 | 89.3 | 8.7 |
| Cool 2014 | RA | RH+ | 92.5 | 6.1 |
| Cool 2014 | RF | RH0 | 104.2 | 5.3 |
| Cool 2014 | RF | RH+ | 103.5 | 8.4 |
| Warm 2015 | Amb | RH0 | 106.0 | 4.4 |
| Warm 2015 | Amb | RH+ | 95.5 | 6.7 |
| Warm 2015 | RA | RH0 | 89.2 | 6.5 |
| Warm 2015 | RA | RH+ | 87.2 | 9.4 |
| Warm 2015 | RF | RH0 | 88.8 | 6.9 |
| Warm 2015 | RF | RH+ | 91.2 | 7.9 |
| Cool 2015 | Amb | RH0 | 139.7 | 5.2 |
| Cool 2015 | Amb | RH+ | 137.2 | 6.9 |
| Cool 2015 | RA | RH0 | 111.7 | 3.7 |
| Cool 2015 | RA | RH+ | 120.8 | 8.8 |
| Cool 2015 | RF | RH0 | 124.3 | 9.6 |
| Cool 2015 | RF | RH+ | 128.0 | 9.4 |

Table A5. Plant responses to rainfall treatments, herbivore addition, and season: dry live +dead mass (g/m^2^) estimates for all plots during October 2014 through April 2015 of the experiment. Amb – Ambient rainfall, RA – Reduced Amount rainfall, RF – Reduced Frequency rainfall, RH_0_ – No scarabs added, RH_+_ - Scarabs added.

| **Total biomass (Oct 2014-April 2015)** | | | | |
| --- | --- | --- | --- | --- |
| **Season** | **Rainfall** | **RH** | **Mass (g/m^2^)** | **SE** |
| Warm | Amb | RH_0_ | 244.9 | 35.1 |
| Warm | Amb | RH_+_ | 271.9 | 15.1 |
| Warm | RA | RH_0_ | 250.1 | 21.6 |
| Warm | RA | RH_+_ | 241.7 | 32.3 |
| Warm | RF | RH_0_ | 239.4 | 18.9 |
| Warm | RF | RH_+_ | 264.4 | 17.8 |
| Cool | Amb | RH_0_ | 69.1 | 11.4 |
| Cool | Amb | RH_+_ | 71.8 | 3.4 |
| Cool | RA | RH_0_ | 87.6 | 8.3 |
| Cool | RA | RH_+_ | 96.3 | 18.2 |
| Cool | RF | RH_0_ | 72.0 | 4.2 |
| Cool | RF | RH_+_ | 85.8 | 7.0 |

Table A6. Plant responses to rainfall treatments and season: dry live +dead mass (g/m^2^) estimates for all plots during October 2014 through April 2015 of the experiment. Amb – Ambient rainfall, RA – Reduced Amount rainfall, RF – Reduced Frequency rainfall, RH_0_ – No scarabs added, RH_+_ - Scarabs added.

| **Total biomass (Oct 2014-April 2015)** | | | |
| --- | --- | --- | --- |
| **Season** | **Rainfall** | **Mass (g/m^2^)** | **SE** |
| Warm | Amb | 258.4 | 18.7 |
| Warm | RA | 245.9 | 18.6 |
| Warm | RF | 251.9 | 13 |
| Cool | Amb | 70.5 | 5.7 |
| Cool | RA | 91.9 | 9.6 |
| Cool | RF | 78.9 | 4.4 |

Table A7. Plant responses to season: dry live +dead mass (g/m^2^) estimates for all plots during October 2014 through April 2015 of the experiment. Amb – Ambient rainfall, RA – Reduced Amount rainfall, RF – Reduced Frequency rainfall, RH_0_ – No scarabs added, RH_+_ - Scarabs added.

| **Total biomass (Oct 2014-April 2015)** | | |
| --- | --- | --- |
| **Season** | **Mass (g/m^2^)** | **SE** |
| Warm | 252.1 | 9.5 |
| Cool | 80.4 | 4.2 |

Table A8. Plant responses to season: dry live +dead mass (g/m^2^) estimates for all plots over time. At cool season 2013, the herbivore treatment was not yet added. Amb – Ambient rainfall, RA – Reduced Amount rainfall, RF – Reduced Frequency rainfall, RH_0_ – No scarabs added, RH_+_ - Scarabs added.

| **Season** | **Rainfall** | **RH** | **Mass (g/m^2^)** | **SE** |
| --- | --- | --- | --- | --- |
| Cool 2013 | Amb | RH_0_ | 281.0 | 32.8 |
| Cool 2013 | Amb | RH_+_ | 320.2 | 25.3 |
| Cool 2013 | RA | RH_0_ | 247.6 | 57.4 |
| Cool 2013 | RA | RH_+_ | 264.0 | 61.6 |
| Cool 2013 | RF | RH_0_ | 244.8 | 45.3 |
| Cool 2013 | RF | RH_+_ | 406.3 | 30.7 |
| Warm 2014 | Amb | RH_0_ | 239.4 | 54.2 |
| Warm 2014 | Amb | RH_+_ | 250.9 | 11.3 |
| Warm 2014 | RA | RH_0_ | 213.6 | 34.9 |
| Warm 2014 | RA | RH_+_ | 201.0 | 27.5 |
| Warm 2014 | RF | RH_0_ | 218.3 | 22.4 |
| Warm 2014 | RF | RH_+_ | 266.2 | 28.8 |
| Cool 2014 | Amb | RH_0_ | 69.1 | 11.4 |
| Cool 2014 | Amb | RH_+_ | 71.8 | 3.4 |
| Cool 2014 | RA | RH_0_ | 87.6 | 8.3 |
| Cool 2014 | RA | RH_+_ | 96.3 | 18.2 |
| Cool 2014 | RF | RH_0_ | 72.0 | 4.2 |
| Cool 2014 | RF | RH_+_ | 85.8 | 7.0 |
| Warm 2015 | Amb | RH_0_ | 250.4 | 20.8 |
| Warm 2015 | Amb | RH_+_ | 292.9 | 25.1 |
| Warm 2015 | RA | RH_0_ | 286.5 | 22.4 |
| Warm 2015 | RA | RH_+_ | 282.4 | 43.3 |
| Warm 2015 | RF | RH_0_ | 260.5 | 22.2 |
| Warm 2015 | RF | RH_+_ | 262.6 | 33.6 |

Table A9. Plant responses to rainfall treatments, herbivore addition, and season: dry live mass (g/m^2^) estimates for all plots during October 2014 through April 2015 of the experiment. Amb – Ambient rainfall, RA – Reduced Amount rainfall, RF – Reduced Frequency rainfall, RH_0_ – No scarabs added, RH_+_ - Scarabs added.

| **Live biomass (Oct 2014-April 2015)** | | | | |
| --- | --- | --- | --- | --- |
| **Season** | **Rainfall** | **RH** | **Mass (g/m^2^)** | **SE** |
| Warm | Amb | RH_0_ | 165.7 | 30.3 |
| Warm | Amb | RH_+_ | 188.0 | 11.1 |
| Warm | RA | RH_0_ | 158.7 | 18 |
| Warm | RA | RH_+_ | 153.7 | 28.1 |
| Warm | RF | RH_0_ | 178.4 | 15.5 |
| Warm | RF | RH_+_ | 184.0 | 15.7 |
| Cool | Amb | RH_0_ | 24.3 | 7.2 |
| Cool | Amb | RH_+_ | 25.0 | 4.2 |
| Cool | RA | RH_0_ | 27.7 | 6.8 |
| Cool | RA | RH_+_ | 35.1 | 10.1 |
| Cool | RF | RH_0_ | 23.8 | 4.9 |
| Cool | RF | RH_+_ | 41.8 | 8.6 |

Table A10. Plant responses to rainfall treatments and season: dry live mass (g/m^2^) estimates for all plots during October 2014 through April 2015 of the experiment. Amb – Ambient rainfall, RA – Reduced Amount rainfall, RF – Reduced Frequency rainfall, RH_0_ – No scarabs added, RH_+_ - Scarabs added.

| **Live biomass (Oct 2014-April 2015)** | | | |
| --- | --- | --- | --- |
| **Season** | **Rainfall** | **Mass (g/m^2^)** | **SE** |
| Warm | Amb | 176.9 | 13.6 |
| Warm | RA | 156.2 | 12.3 |
| Warm | RF | 181.2 | 11.9 |
| Cool | Amb | 24.6 | 4.0 |
| Cool | RA | 31.4 | 5.9 |
| Cool | RF | 32.8 | 5.5 |

Table A11. Plant responses to season: dry live mass (g/m^2^) estimates for all plots during October 2014 through April 2015 of the experiment. Amb – Ambient rainfall, RA – Reduced Amount rainfall, RF – Reduced Frequency rainfall, RH_0_ – No scarabs added, RH_+_ - Scarabs added.

| **Live biomass (Oct 2014-April 2015)** | | |
| --- | --- | --- |
| **Season** | **Mass (g/m^2^)** | **SE** |
| Warm | 171.4 | 8.2 |
| Cool | 29.6 | 3 |

**Table A12. Plant responses to scarab addition: dry live mass (g/m^2^) estimates during October 2013 (pre-scarab) through April 2015 of the experiment. Amb – Ambient rainfall, RA – Reduced Amount rainfall, RF – Reduced Frequency rainfall, RH_0_ – No scarabs added, RH_+_ - Scarabs added.**

| **Season** | **RH** | **Mass (g/m^2^)** | **SE** |
| --- | --- | --- | --- |
| Pre-scarab | RH_0_ | 134.4 | 11.7 |
| Pre-scarab | RH_+_ | 246.8 | 24.0 |
| Warm | RH_0_ | 167.6 | 10.3 |
| Warm | RH_+_ | 175.2 | 10.4 |
| Cool | RH_0_ | 25.2 | 3.5 |
| Cool | RH_+_ | 34.0 | 4.7 |

Table A13. Plant responses to season: dry live (g/m^2^) estimates for all plots over time. At cool season 2013, the herbivore treatment was not yet added. Amb – Ambient rainfall, RA – Reduced Amount rainfall, RF – Reduced Frequency rainfall, RH_0_ – No scarabs added, RH_+_ - Scarabs added.

| **Season** | **Rainfall** | **RH** | **Mass (g/m^2^)** | **SE** |
| --- | --- | --- | --- | --- |
| Cool 2013 | Amb | RH_0_ | 149.1 | 21.2 |
| Cool 2013 | Amb | RH_+_ | 227.8 | 25.4 |
| Cool 2013 | RA | RH_0_ | 127.9 | 22.2 |
| Cool 2013 | RA | RH_+_ | 191.4 | 49.7 |
| Cool 2013 | RF | RH_0_ | 126.1 | 19.6 |
| Cool 2013 | RF | RH_+_ | 321.3 | 31.1 |
| Warm 2014 | Amb | RH_0_ | 197.5 | 47.2 |
| Warm 2014 | Amb | RH_+_ | 205.5 | 10.0 |
| Warm 2014 | RA | RH_0_ | 170.7 | 21.9 |
| Warm 2014 | RA | RH_+_ | 156.5 | 21.8 |
| Warm 2014 | RF | RH_0_ | 171.8 | 17.6 |
| Warm 2014 | RF | RH_+_ | 217.6 | 30.3 |
| Cool 2014 | Amb | RH_0_ | 24.3 | 7.2 |
| Cool 2014 | Amb | RH_+_ | 25.0 | 4.2 |
| Cool 2014 | RA | RH_0_ | 27.7 | 6.8 |
| Cool 2014 | RA | RH_+_ | 35.1 | 10.1 |
| Cool 2014 | RF | RH_0_ | 23.8 | 4.9 |
| Cool 2014 | RF | RH_+_ | 41.8 | 8.6 |
| Warm 2015 | Amb | RH_0_ | 134.0 | 15.3 |
| Warm 2015 | Amb | RH_+_ | 170.4 | 15.3 |
| Warm 2015 | RA | RH_0_ | 146.7 | 18.0 |
| Warm 2015 | RA | RH_+_ | 151.0 | 37.6 |
| Warm 2015 | RF | RH_0_ | 185.1 | 19.0 |
| Warm 2015 | RF | RH_+_ | 150.5 | 23.1 |

Table A14. Plant responses to rainfall treatments, herbivore addition, and season: dry dead mass (g/m^2^) estimates for all plots during October 2014 through April 2015 of the experiment. Amb – Ambient rainfall, RA – Reduced Amount rainfall, RF – Reduced Frequency rainfall, RH_0_ – No scarabs added, RH_+_ - Scarabs added.

| **Dead biomass (Oct 2014-April 2015)** | | | | |
| --- | --- | --- | --- | --- |
| **Season** | **Rainfall** | **RH** | **Mass (g/m^2^)** | **SE** |
| Warm | Amb | RH_0_ | 79.1 | 11.9 |
| Warm | Amb | RH_+_ | 83.9 | 8.9 |
| Warm | RA | RH_0_ | 91.4 | 5.3 |
| Warm | RA | RH_+_ | 87.9 | 12.1 |
| Warm | RF | RH_0_ | 61.0 | 4.9 |
| Warm | RF | RH_+_ | 80.4 | 14.1 |
| Cool | Amb | RH_0_ | 44.9 | 6.3 |
| Cool | Amb | RH_+_ | 46.8 | 6.5 |
| Cool | RA | RH_0_ | 59.8 | 4.1 |
| Cool | RA | RH_+_ | 61.2 | 12.1 |
| Cool | RF | RH_0_ | 48.2 | 3.8 |
| Cool | RF | RH_+_ | 44.0 | 4.4 |

Table A15. Plant responses to rainfall treatments and season: dry dead mass (g/m^2^) estimates for all plots during October 2014 through April 2015 of the experiment. Amb – Ambient rainfall, RA – Reduced Amount rainfall, RF – Reduced Frequency rainfall, RH_0_ – No scarabs added, RH_+_ - Scarabs added.

| **Dead biomass (Oct 2014-April 2015)** | | | |
| --- | --- | --- | --- |
| **Season** | **Rainfall** | **Mass (g/m^2^)** | **SE** |
| Warm | Amb | 81.5 | 7.1 |
| Warm | RA | 89.7 | 6.3 |
| Warm | RF | 70.7 | 7.7 |
| Cool | Amb | 45.8 | 4.3 |
| Cool | RA | 60.5 | 6.1 |
| Cool | RF | 46.1 | 2.8 |

Table A16. Plant responses to season: dry dead mass (g/m^2^) estimates for all plots during October 2014 through April 2015 of the experiment. Amb – Ambient rainfall, RA – Reduced Amount rainfall, RF – Reduced Frequency rainfall, RH_0_ – No scarabs added, RH_+_ - Scarabs added.

| **Dead biomass (Oct 2014-April 2015)** | | |
| --- | --- | --- |
| **Season** | **Mass (g/m^2^)** | **SE** |
| Warm | 80.6 | 4.2 |
| Cool | 50.8 | 2.8 |

Table A17. Plant responses to season: dry dead mass (g/m^2^) estimates for all plots over time. At cool season 2013, the herbivore treatment was not yet added. Amb – Ambient rainfall, RA – Reduced Amount rainfall, RF – Reduced Frequency rainfall, RH_0_ – No scarabs added, RH_+_ - Scarabs added.

| **Season** | **Rainfall** | **RH** | **Mass (g/m^2^)** | **SE** |
| --- | --- | --- | --- | --- |
| Cool 2013 | Amb | RH_0_ | 131.9 | 16.4 |
| Cool 2013 | Amb | RH_+_ | 92.5 | 1.7 |
| Cool 2013 | RA | RH_0_ | 119.6 | 37.3 |
| Cool 2013 | RA | RH_+_ | 72.6 | 23.4 |
| Cool 2013 | RF | RH_0_ | 118.7 | 26.9 |
| Cool 2013 | RF | RH_+_ | 85.0 | 18.3 |
| Warm 2014 | Amb | RH_0_ | 41.9 | 8.2 |
| Warm 2014 | Amb | RH_+_ | 45.4 | 5.4 |
| Warm 2014 | RA | RH_0_ | 42.9 | 14.9 |
| Warm 2014 | RA | RH_+_ | 44.5 | 8.9 |
| Warm 2014 | RF | RH_0_ | 46.5 | 5.4 |
| Warm 2014 | RF | RH_+_ | 48.6 | 5.4 |
| Cool 2014 | Amb | RH_0_ | 44.9 | 6.3 |
| Cool 2014 | Amb | RH_+_ | 46.8 | 6.5 |
| Cool 2014 | RA | RH_0_ | 59.8 | 4.1 |
| Cool 2014 | RA | RH_+_ | 61.2 | 12.1 |
| Cool 2014 | RF | RH_0_ | 48.2 | 3.8 |
| Cool 2014 | RF | RH_+_ | 44.0 | 4.4 |
| Warm 2015 | Amb | RH_0_ | 116.4 | 21.3 |
| Warm 2015 | Amb | RH_+_ | 122.5 | 15.3 |
| Warm 2015 | RA | RH_0_ | 139.9 | 11.7 |
| Warm 2015 | RA | RH_+_ | 131.4 | 20.8 |
| Warm 2015 | RF | RH_0_ | 75.5 | 7.2 |
| Warm 2015 | RF | RH_+_ | 112.1 | 24.6 |

Table A18. Dry biomass from root-ingrowth core samples over the course of the experiment. At standing crop, the herbivore treatment was not yet added. Amb – Ambient rainfall, RA – Reduced Amount rainfall, RF – Reduced Frequency rainfall, RH_0_ – No scarabs added, RH_+_ - Scarabs added.

| **Season** | **Rainfall** | **RH** | **Dry Mass** | **SE** |
| --- | --- | --- | --- | --- |
| Standing Crop | Amb | RH_0_ | 1.191 | 0.154 |
| Standing Crop | Amb | RH_+_ | 1.181 | 0.122 |
| Standing Crop | RA | RH_0_ | 1.054 | 0.144 |
| Standing Crop | RA | RH_+_ | 1.289 | 0.101 |
| Standing Crop | RF | RH_0_ | 0.822 | 0.065 |
| Standing Crop | RF | RH_+_ | 0.764 | 0.149 |
| Warm Season 2014 | Amb | RH_0_ | 0.231 | 0.028 |
| Warm Season 2014 | Amb | RH_+_ | 0.245 | 0.010 |
| Warm Season 2014 | RA | RH_0_ | 0.225 | 0.023 |
| Warm Season 2014 | RA | RH_+_ | 0.250 | 0.008 |
| Warm Season 2014 | RF | RH_0_ | 0.172 | 0.016 |
| Warm Season 2014 | RF | RH_+_ | 0.235 | 0.042 |
| Cool Season 2015 | Amb | RH_0_ | 0.081 | 0.014 |
| Cool Season 2015 | Amb | RH_+_ | 0.092 | 0.009 |
| Cool Season 2015 | RA | RH_0_ | 0.074 | 0.015 |
| Cool Season 2015 | RA | RH_+_ | 0.070 | 0.017 |
| Cool Season 2015 | RF | RH_0_ | 0.050 | 0.010 |
| Cool Season 2015 | RF | RH_+_ | 0.071 | 0.019 |
| Warm Season 2015 | Amb | RH_0_ | 0.031 | 0.004 |
| Warm Season 2015 | Amb | RH_+_ | 0.029 | 0.004 |
| Warm Season 2015 | RA | RH_0_ | 0.037 | 0.005 |
| Warm Season 2015 | RA | RH_+_ | 0.029 | 0.005 |
| Warm Season 2015 | RF | RH_0_ | 0.029 | 0.002 |
| Warm Season 2015 | RF | RH_+_ | 0.041 | 0.005 |

Table A19. Dry biomass from root-ingrowth core samples over the course of the experiment normalized (due to diminishing returns: per season treatments/per season amb/RH_0_). At standing crop, the herbivore treatment was not yet added. Amb – Ambient rainfall, RA – Reduced Amount rainfall, RF – Reduced Frequency rainfall, RH_0_ – No scarabs added, RH_+_ - Scarabs added.

| **Season** | **Year** | **Rainfall** | **RH** | **Normalized Mass** | **SE** |
| --- | --- | --- | --- | --- | --- |
| Standing Crop | 2013 | RA | RH_0_ | -0.115 | 0.121 |
| Standing Crop | 2013 | RA | RH_+_ | 0.091 | 0.085 |
| Standing Crop | 2013 | RF | RH_0_ | -0.310 | 0.055 |
| Standing Crop | 2013 | RF | RH_+_ | -0.353 | 0.126 |
| Warm Season | 2014 | RA | RH_0_ | -0.027 | 0.099 |
| Warm Season | 2014 | RA | RH_+_ | 0.018 | 0.032 |
| Warm Season | 2014 | RF | RH_0_ | -0.256 | 0.071 |
| Warm Season | 2014 | RF | RH_+_ | -0.042 | 0.171 |
| Cool Season | 2015 | RA | RH_0_ | -0.084 | 0.189 |
| Cool Season | 2015 | RA | RH_+_ | -0.238 | 0.184 |
| Cool Season | 2015 | RF | RH_0_ | -0.385 | 0.122 |
| Cool Season | 2015 | RF | RH_+_ | -0.228 | 0.209 |
| Warm Season | 2015 | RA | RH_0_ | 0.211 | 0.160 |
| Warm Season | 2015 | RA | RH_+_ | -0.001 | 0.179 |
| Warm Season | 2015 | RF | RH_0_ | -0.046 | 0.068 |
| Warm Season | 2015 | RF | RH_+_ | 0.428 | 0.174 |

Table A20. Biomass-based C_3_/C_4_ ratios. At cool season 2013, the herbivore treatment was not yet added. Amb – Ambient rainfall, RA – Reduced Amount rainfall, RF – Reduced Frequency rainfall, RH_0_ – No scarabs added, RH_+_ - Scarabs added.

| **Season** | **Rainfall** | **RH** | **C_3_/C_4_** | **SE** |
| --- | --- | --- | --- | --- |
| Cool 2013 | Amb | RH_0_ | 0.619 | 0.109 |
| Cool 2013 | Amb | RH_+_ | 1.114 | 0.327 |
| Cool 2013 | RA | RH_0_ | 0.736 | 0.257 |
| Cool 2013 | RA | RH_+_ | 0.818 | 0.186 |
| Cool 2013 | RF | RH_0_ | 0.573 | 0.161 |
| Cool 2013 | RF | RH_+_ | 0.816 | 0.297 |
| Warm 2014 | Amb | RH_0_ | 0.195 | 0.122 |
| Warm 2014 | Amb | RH_+_ | 0.051 | 0.015 |
| Warm 2014 | RA | RH_0_ | 0.04 | 0.019 |
| Warm 2014 | RA | RH_+_ | 0.053 | 0.015 |
| Warm 2014 | RF | RH_0_ | 0.139 | 0.117 |
| Warm 2014 | RF | RH_+_ | 0.12 | 0.074 |
| Cool 2014 | Amb | RH_0_ | 0.901 | 0.493 |
| Cool 2014 | Amb | RH_+_ | 0.665 | 0.231 |
| Cool 2014 | RA | RH_0_ | 0.098 | 0.022 |
| Cool 2014 | RA | RH_+_ | 0.411 | 0.254 |
| Cool 2014 | RF | RH_0_ | 0.421 | 0.212 |
| Cool 2014 | RF | RH_+_ | 0.467 | 0.098 |
| Warm 2015 | Amb | RH_0_ | 0.13 | 0.063 |
| Warm 2015 | Amb | RH_+_ | 0.029 | 0.005 |
| Warm 2015 | RA | RH_0_ | 0.032 | 0.016 |
| Warm 2015 | RA | RH_+_ | 0.02 | 0.005 |
| Warm 2015 | RF | RH_0_ | 0.142 | 0.075 |
| Warm 2015 | RF | RH_+_ | 0.089 | 0.02 |

Table A21. Cover-based C_3_/C_4_ ratios. At cool season 2013, the herbivore treatment was not yet added. Amb – Ambient rainfall, RA – Reduced Amount rainfall, RF – Reduced Frequency rainfall, RH_0_ – No scarabs added, RH_+_ - Scarabs added.

| **Season** | **Rainfall** | **RH** | **C_3_/C_4_** | **SE** |
| --- | --- | --- | --- | --- |
| Cool 2013 | Amb | RH_0_ | 2.153 | 0.473 |
| Cool 2013 | Amb | RH_+_ | 1.697 | 0.226 |
| Cool 2013 | RA | RH_0_ | 1.800 | 0.385 |
| Cool 2013 | RA | RH_+_ | 1.989 | 0.200 |
| Cool 2013 | RF | RH_0_ | 1.562 | 0.163 |
| Cool 2013 | RF | RH_+_ | 2.146 | 0.410 |
| Warm 2014 | Amb | RH_0_ | 0.303 | 0.086 |
| Warm 2014 | Amb | RH_+_ | 0.255 | 0.058 |
| Warm 2014 | RA | RH_0_ | 0.170 | 0.062 |
| Warm 2014 | RA | RH_+_ | 0.287 | 0.067 |
| Warm 2014 | RF | RH_0_ | 0.317 | 0.059 |
| Warm 2014 | RF | RH_+_ | 0.404 | 0.077 |
| Cool 2014 | Amb | RH_0_ | 1.210 | 0.198 |
| Cool 2014 | Amb | RH_+_ | 1.036 | 0.212 |
| Cool 2014 | RA | RH_0_ | 0.463 | 0.090 |
| Cool 2014 | RA | RH_+_ | 0.923 | 0.197 |
| Cool 2014 | RF | RH_0_ | 0.856 | 0.159 |
| Cool 2014 | RF | RH_+_ | 1.293 | 0.270 |
| Warm 2015 | Amb | RH_0_ | 0.436 | 0.102 |
| Warm 2015 | Amb | RH_+_ | 0.375 | 0.129 |
| Warm 2015 | RA | RH_0_ | 0.473 | 0.136 |
| Warm 2015 | RA | RH_+_ | 0.290 | 0.05 |
| Warm 2015 | RF | RH_0_ | 0.341 | 0.058 |
| Warm 2015 | RF | RH_+_ | 0.583 | 0.100 |
| Cool 2015 | Amb | RH_0_ | 1.488 | 0.311 |
| Cool 2015 | Amb | RH_+_ | 1.827 | 0.518 |
| Cool 2015 | RA | RH_0_ | 1.105 | 0.209 |
| Cool 2015 | RA | RH_+_ | 1.612 | 0.337 |
| Cool 2015 | RF | RH_0_ | 1.440 | 0.398 |
| Cool 2015 | RF | RH_+_ | 2.443 | 0.532 |

Table A22. Biomass-based diversity (Shannon-Wiener *H*). At cool season 2013, the herbivore treatment was not yet added. Amb – Ambient rainfall, RA – Reduced Amount rainfall, RF – Reduced Frequency rainfall, RH_0_ – No scarabs added, RH_+_ - Scarabs added.

| **Season** | **Rainfall** | **RH** | **Shannon-Wiener (***H***)** | **SE** |
| --- | --- | --- | --- | --- |
| Cool 2013 | Amb | RH_0_ | 1.576 | 0.088 |
| Cool 2013 | Amb | RH_+_ | 1.916 | 0.036 |
| Cool 2013 | RA | RH_0_ | 1.585 | 0.144 |
| Cool 2013 | RA | RH_+_ | 1.826 | 0.066 |
| Cool 2013 | RF | RH_0_ | 1.591 | 0.08 |
| Cool 2013 | RF | RH_+_ | 1.672 | 0.109 |
| Warm 2014 | Amb | RH_0_ | 1.763 | 0.1 |
| Warm 2014 | Amb | RH_+_ | 1.582 | 0.098 |
| Warm 2014 | RA | RH_0_ | 1.381 | 0.151 |
| Warm 2014 | RA | RH_+_ | 1.532 | 0.033 |
| Warm 2014 | RF | RH_0_ | 1.504 | 0.081 |
| Warm 2014 | RF | RH_+_ | 1.229 | 0.083 |
| Cool 2014 | Amb | RH_0_ | 1.085 | 0.141 |
| Cool 2014 | Amb | RH_+_ | 1.159 | 0.145 |
| Cool 2014 | RA | RH_0_ | 0.889 | 0.08 |
| Cool 2014 | RA | RH_+_ | 0.965 | 0.099 |
| Cool 2014 | RF | RH_0_ | 0.983 | 0.109 |
| Cool 2014 | RF | RH_+_ | 1.25 | 0.11 |
| Warm 2015 | Amb | RH_0_ | 1.557 | 0.123 |
| Warm 2015 | Amb | RH_+_ | 1.459 | 0.118 |
| Warm 2015 | RA | RH_0_ | 1.293 | 0.05 |
| Warm 2015 | RA | RH_+_ | 1.32 | 0.083 |
| Warm 2015 | RF | RH_0_ | 1.571 | 0.089 |
| Warm 2015 | RF | RH_+_ | 1.33 | 0.139 |

Table A23. Biomass-based evenness. At cool season 2013, the herbivore treatment was not yet added. Amb – Ambient rainfall, RA – Reduced Amount rainfall, RF – Reduced Frequency rainfall, RH_0_ – No scarabs added, RH_+_ - Scarabs added.

| **Season** | **Rainfall** | **RH** | **Evenness** | **SE** |
| --- | --- | --- | --- | --- |
| Cool 2013 | Amb | RH_0_ | 0.672 | 0.034 |
| Cool 2013 | Amb | RH_+_ | 0.788 | 0.014 |
| Cool 2013 | RA | RH_0_ | 0.662 | 0.055 |
| Cool 2013 | RA | RH_+_ | 0.787 | 0.027 |
| Cool 2013 | RF | RH_0_ | 0.661 | 0.027 |
| Cool 2013 | RF | RH_+_ | 0.762 | 0.029 |
| Warm 2014 | Amb | RH_0_ | 0.804 | 0.03 |
| Warm 2014 | Amb | RH_+_ | 0.693 | 0.031 |
| Warm 2014 | RA | RH_0_ | 0.657 | 0.055 |
| Warm 2014 | RA | RH_+_ | 0.746 | 0.036 |
| Warm 2014 | RF | RH_0_ | 0.697 | 0.027 |
| Warm 2014 | RF | RH_+_ | 0.614 | 0.061 |
| Cool 2014 | Amb | RH_0_ | 0.464 | 0.052 |
| Cool 2014 | Amb | RH_+_ | 0.491 | 0.054 |
| Cool 2014 | RA | RH_0_ | 0.433 | 0.048 |
| Cool 2014 | RA | RH_+_ | 0.456 | 0.035 |
| Cool 2014 | RF | RH_0_ | 0.464 | 0.044 |
| Cool 2014 | RF | RH_+_ | 0.54 | 0.038 |
| Warm 2015 | Amb | RH_0_ | 0.666 | 0.048 |
| Warm 2015 | Amb | RH_+_ | 0.62 | 0.042 |
| Warm 2015 | RA | RH_0_ | 0.617 | 0.037 |
| Warm 2015 | RA | RH_+_ | 0.624 | 0.039 |
| Warm 2015 | RF | RH_0_ | 0.727 | 0.032 |
| Warm 2015 | RF | RH_+_ | 0.612 | 0.06 |

Table A24. Cover-based diversity (Shannon-Wiener *H*). At cool season 2013, the herbivore treatment was not yet added. Amb – Ambient rainfall, RA – Reduced Amount rainfall, RF – Reduced Frequency rainfall, RH_0_ – No scarabs added, RH_+_ - Scarabs added.

| **Season** | **Rainfall** | **RH** | **Shannon-Wiener (***H***)** | **SE** |
| --- | --- | --- | --- | --- |
| Cool 2013 | Amb | RH_0_ | 2.375 | 0.039 |
| Cool 2013 | Amb | RH_+_ | 2.333 | 0.048 |
| Cool 2013 | RA | RH_0_ | 2.273 | 0.066 |
| Cool 2013 | RA | RH_+_ | 2.341 | 0.046 |
| Cool 2013 | RF | RH_0_ | 2.17 | 0.063 |
| Cool 2013 | RF | RH_+_ | 2.365 | 0.069 |
| Warm 2014 | Amb | RH_0_ | 2.046 | 0.07 |
| Warm 2014 | Amb | RH_+_ | 1.884 | 0.132 |
| Warm 2014 | RA | RH_0_ | 1.601 | 0.103 |
| Warm 2014 | RA | RH_+_ | 1.681 | 0.184 |
| Warm 2014 | RF | RH_0_ | 1.619 | 0.11 |
| Warm 2014 | RF | RH_+_ | 1.729 | 0.07 |
| Cool 2014 | Amb | RH_0_ | 2.438 | 0.055 |
| Cool 2014 | Amb | RH_+_ | 2.434 | 0.047 |
| Cool 2014 | RA | RH_0_ | 2.312 | 0.081 |
| Cool 2014 | RA | RH_+_ | 2.303 | 0.048 |
| Cool 2014 | RF | RH_0_ | 2.248 | 0.099 |
| Cool 2014 | RF | RH_+_ | 2.207 | 0.048 |
| Warm 2015 | Amb | RH_0_ | 2.039 | 0.076 |
| Warm 2015 | Amb | RH_+_ | 2.112 | 0.026 |
| Warm 2015 | RA | RH_0_ | 1.972 | 0.076 |
| Warm 2015 | RA | RH_+_ | 2.008 | 0.069 |
| Warm 2015 | RF | RH_0_ | 2.089 | 0.094 |
| Warm 2015 | RF | RH_+_ | 2.07 | 0.075 |
| Cool 2015 | Amb | RH_0_ | 2.429 | 0.074 |
| Cool 2015 | Amb | RH_+_ | 2.482 | 0.073 |
| Cool 2015 | RA | RH_0_ | 2.319 | 0.096 |
| Cool 2015 | RA | RH_+_ | 2.364 | 0.04 |
| Cool 2015 | RF | RH_0_ | 2.373 | 0.076 |
| Cool 2015 | RF | RH_+_ | 2.368 | 0.085 |

Table A25. Cover-based evenness. At cool season 2013, the herbivore treatment was not yet added. Amb – Ambient rainfall, RA – Reduced Amount rainfall, RF – Reduced Frequency rainfall, RH_0_ – No scarabs added, RH_+_ - Scarabs added.

| **Season** | **Rainfall** | **RH** | **Evenness** | **SE** |
| --- | --- | --- | --- | --- |
| Cool 2013 | Amb | RH_0_ | 0.887 | 0.014 |
| Cool 2013 | Amb | RH_+_ | 0.895 | 0.018 |
| Cool 2013 | RA | RH_0_ | 0.863 | 0.016 |
| Cool 2013 | RA | RH_+_ | 0.891 | 0.01 |
| Cool 2013 | RF | RH_0_ | 0.871 | 0.028 |
| Cool 2013 | RF | RH_+_ | 0.892 | 0.008 |
| Warm 2014 | Amb | RH_0_ | 0.851 | 0.028 |
| Warm 2014 | Amb | RH_+_ | 0.833 | 0.03 |
| Warm 2014 | RA | RH_0_ | 0.791 | 0.025 |
| Warm 2014 | RA | RH_+_ | 0.817 | 0.047 |
| Warm 2014 | RF | RH_0_ | 0.818 | 0.028 |
| Warm 2014 | RF | RH_+_ | 0.82 | 0.02 |
| Cool 2014 | Amb | RH_0_ | 0.863 | 0.012 |
| Cool 2014 | Amb | RH_+_ | 0.882 | 0.014 |
| Cool 2014 | RA | RH_0_ | 0.876 | 0.014 |
| Cool 2014 | RA | RH_+_ | 0.873 | 0.012 |
| Cool 2014 | RF | RH_0_ | 0.852 | 0.017 |
| Cool 2014 | RF | RH_+_ | 0.889 | 0.014 |
| Warm 2015 | Amb | RH_0_ | 0.847 | 0.01 |
| Warm 2015 | Amb | RH_+_ | 0.855 | 0.009 |
| Warm 2015 | RA | RH_0_ | 0.841 | 0.014 |
| Warm 2015 | RA | RH_+_ | 0.873 | 0.011 |
| Warm 2015 | RF | RH_0_ | 0.824 | 0.027 |
| Warm 2015 | RF | RH_+_ | 0.842 | 0.008 |
| Cool 2015 | Amb | RH_0_ | 0.872 | 0.009 |
| Cool 2015 | Amb | RH_+_ | 0.884 | 0.015 |
| Cool 2015 | RA | RH_0_ | 0.874 | 0.013 |
| Cool 2015 | RA | RH_+_ | 0.894 | 0.015 |
| Cool 2015 | RF | RH_0_ | 0.869 | 0.008 |
| Cool 2015 | RF | RH_+_ | 0.884 | 0.008 |

**Table A26. Soil moisture (%) repeated measures linear mixed effects model (fixed: season*rain-treatment + rainfall anova) table.**

| **Variable** | **Chisq** | **Df** | **Pr(>Chisq)** |
| --- | --- | --- | --- |
| Rainfall | 6.417553 | 2 | 0.0404 |
| Season | 32.29909 | 1 | <0.001 |
| total | 196.1855 | 1 | <0.001 |

**Table A27. Plant cover permanova analysis.**

| **Variable** | **Df** | **SumsOfSqs** | **MeanSqs** | **F.Model** | **R^2^** | **Pr(>F)** |
| --- | --- | --- | --- | --- | --- | --- |
| Rainfall | 2 | 6469.424 | 3234.712 | 13.582 | 0.174 | 0.001 |
| RH | 1 | 8.681 | 8.681 | 0.036 | 0 | 0.856 |
| Season | 1 | 15694.01 | 15694.01 | 65.897 | 0.422 | 0.001 |
| Rainfall: RH | 2 | 18.132 | 9.066 | 0.038 | 0 | 0.963 |
| Rainfall: Season | 2 | 560.632 | 280.316 | 1.177 | 0.015 | 0.288 |
| RH: Season | 1 | 102.722 | 102.722 | 0.431 | 0.003 | 0.543 |
| Rainfall: RH: Season | 2 | 76.34 | 38.17 | 0.16 | 0.002 | 0.853 |
| Residuals | 60 | 14289.5 | 238.158 | NA | 0.384 | NA |
| Total | 71 | 37219.44 | NA | NA | 1 | NA |

**Table A28. Plant cover permanova analysis pre-scarab addition.**

| **Variable** | **Df** | **SumsOfSqs** | **MeanSqs** | **F.Model** | **R^2^** | **Pr(>F)** |
| --- | --- | --- | --- | --- | --- | --- |
| Rainfall | 2 | 1228.5 | 614.25 | 1.37984 | 0.07338 | 0.267 |
| RH | 1 | 380.3 | 380.25 | 0.85419 | 0.02271 | 0.356 |
| Rainfall:RH | 2 | 1777.2 | 888.58 | 1.99609 | 0.10616 | 0.154 |
| Residuals | 30 | 13354.8 | 445.16 | 0.79774 |  |  |
| Total | 35 | 16740.8 | 1 |  |  |  |

**Table A29. Plant total (live + dead) biomass permanova analysis.**

| **Variable** | **Df** | **SumsOfSqs** | **MeanSqs** | **F.Model** | **R^2^** | **Pr(>F)** |
| --- | --- | --- | --- | --- | --- | --- |
| Rainfall | 2 | 265.87 | 132.935 | 0.062 | 0 | 0.937 |
| RH | 1 | 2371.911 | 2371.911 | 1.115 | 0.004 | 0.298 |
| Season | 1 | 530211.7 | 530211.7 | 249.171 | 0.795 | 0.001 |
| Rainfall:RH | 2 | 1209.85 | 604.925 | 0.284 | 0.002 | 0.747 |
| Rainfall:Season | 2 | 3483.844 | 1741.922 | 0.819 | 0.005 | 0.477 |
| RH:Season | 1 | 168.401 | 168.401 | 0.079 | 0 | 0.8 |
| Rainfall:RH:Season | 2 | 1349.989 | 674.995 | 0.317 | 0.002 | 0.737 |
| Residuals | 60 | 127674 | 2127.9 | NA | 0.191 | NA |
| Total | 71 | 666735.6 | NA | NA | 1 | NA |

**Table A30. Plant total (live + dead) biomass permanova analysis pre-scarab addition.**

| **Variable** | **Df** | **SumsOfSqs** | **MeanSqs** | **F.Model** | **R^2^** | **Pr(>F)** |
| --- | --- | --- | --- | --- | --- | --- |
| Rainfall | 2 | 0.004113 | 0.002057 | 1.19419 | 0.06748 | 0.331 |
| RH | 1 | 0.00269 | 0.00269 | 1.56218 | 0.04414 | 0.213 |
| Rainfall:RH | 2 | 0.002483 | 0.001241 | 0.72085 | 0.04074 | 0.462 |
| Residuals | 30 | 0.051663 | 0.001722 | 0.84764 |  |  |
| Total | 35 | 0.060949 | 1 |  |  |  |

**Table A31. Plant live biomass permanova analysis.**

| **Variable** | **Df** | **SumsOfSqs** | **MeanSqs** | **F.Model** | **R^2^** | **Pr(>F)** |
| --- | --- | --- | --- | --- | --- | --- |
| Rainfall | 2 | 0.349 | 0.174 | 0.766 | 0.004 | 0.449 |
| RH | 1 | 0.475 | 0.475 | 2.089 | 0.006 | 0.134 |
| Season | 1 | 64.246 | 64.246 | 282.482 | 0.806 | 0.001 |
| Rainfall:RH | 2 | 0.227 | 0.113 | 0.499 | 0.003 | 0.61 |
| Rainfall:Season | 2 | 0.262 | 0.131 | 0.577 | 0.003 | 0.579 |
| RH:Season | 1 | 0.218 | 0.218 | 0.957 | 0.003 | 0.364 |
| Rainfall:RH:Season | 2 | 0.25 | 0.125 | 0.55 | 0.003 | 0.565 |
| Residuals | 60 | 13.646 | 0.227 | NA | 0.171 | NA |
| Total | 71 | 79.672 | NA | NA | 1 | NA |

**Table A32. Plant live biomass permanova analysis pre-scarab addition.**

| **Variable** | **Df** | **SumsOfSqs** | **MeanSqs** | **F.Model** | **R^2^** | **Pr(>F)** |
| --- | --- | --- | --- | --- | --- | --- |
| Rainfall | 2 | 0.8318 | 0.41588 | 1.6982 | 0.06883 | 0.185 |
| RH | 1 | 3.079 | 3.07903 | 12.5729 | 0.2548 | 0.001 |
| Rainfall:RH | 2 | 0.8267 | 0.41333 | 1.6878 | 0.06841 | 0.189 |
| Residuals | 30 | 7.3468 | 0.24489 | 0.60797 |  |  |
| Total | 35 | 12.0843 | 1 |  |  |  |

**Table A33. Plant dead biomass permanova analysis.**

| **Variable** | **Df** | **SumsOfSqs** | **MeanSqs** | **F.Model** | **R^2^** | **Pr(>F)** |
| --- | --- | --- | --- | --- | --- | --- |
| Rainfall | 2 | 3492.696 | 1746.348 | 3.89 | 0.073 | 0.017 |
| RH | 1 | 196.096 | 196.096 | 0.437 | 0.004 | 0.524 |
| Season | 1 | 15993.608 | 15993.608 | 35.662 | 0.333 | 0.001 |
| Rainfall:RH | 2 | 222.272 | 111.136 | 0.248 | 0.005 | 0.778 |
| Rainfall:Season | 2 | 376.583 | 188.292 | 0.42 | 0.008 | 0.676 |
| RH:Season | 1 | 234.66 | 234.66 | 0.523 | 0.005 | 0.478 |
| Rainfall:RH:Season | 2 | 648.448 | 324.224 | 0.723 | 0.013 | 0.488 |
| Residuals | 60 | 26908.496 | 448.475 | NA | 0.56 | NA |
| Total | 71 | 48072.858 | NA | NA | 1 | NA |

**Table A34. Plant dead biomass permanova analysis.**

| **Variable** | **Df** | **SumsOfSqs** | **MeanSqs** | **F.Model** | **R^2^** | **Pr(>F)** |
| --- | --- | --- | --- | --- | --- | --- |
| Rainfall | 2 | 1594 | 797 | 0.2439 | 0.01394 | 0.802 |
| RH | 1 | 14441 | 14440.8 | 4.4194 | 0.12631 | 0.058 |
| Rainfall:RH | 2 | 269 | 134.6 | 0.0412 | 0.00235 | 0.961 |
| Residuals | 30 | 98028 | 3267.6 | 0.8574 |  |  |
| Total | 35 | 114332 | 1 |  |  |  |

**Table A35. Root biomass repeated measures ANOVA (Type III).**

| **Variable** | **Chisq** | **Df** | **Pr(>Chisq)** |
| --- | --- | --- | --- |
| (Intercept) | 10.4492 | 1 | 0.001227 |
| Rainfall | 7.0528 | 2 | 0.029411 |
| RH | 2.1351 | 1 | 0.143957 |
| Season | 0.7574 | 1 | 0.384135 |
| Standing Crop | 8.0673 | 1 | 0.004507 |
| Rainfall:RH | 3.6314 | 2 | 0.162723 |
| Rainfall:Season | 2.6716 | 2 | 0.262951 |
| RH:Season | 0.0047 | 1 | 0.945072 |
| Rainfall:RH:Season | 0.1409 | 2 | 0.931983 |

**Table A36. Root biomass repeated measures ANOVA (Type III) pre-scarab addition.**

| **Variable** | **SumsOfSqs** | **Df** | **F value** | **Pr(>F)** |
| --- | --- | --- | --- | --- |
| (Intercept) | 0.028 | 1 | 0.0264 | 0.8719 |
| Rainfall | 10.578 | 2 | 5.0087 | 0.01328 |
| RH | 0.015 | 1 | 0.0143 | 0.90567 |
| Rainfall:RH | 1.668 | 2 | 0.7899 | 0.46309 |
| Residuals | 31.679 | 30 |  |  |

**Table A37. Cover C3:C4 ratio permanova analysis.**

| **Variable** | **Df** | **SumsOfSqs** | **MeanSqs** | **F.Model** | **R^2^** | **Pr(>F)** |
| --- | --- | --- | --- | --- | --- | --- |
| Rainfall | 2 | 0.459 | 0.229 | 3.584 | 0.025 | 0.034 |
| RH | 1 | 0.301 | 0.301 | 4.708 | 0.016 | 0.038 |
| Season | 1 | 8.537 | 8.537 | 133.368 | 0.465 | 0.001 |
| Rainfall:RH | 2 | 0.257 | 0.128 | 2.005 | 0.014 | 0.143 |
| Rainfall:Season | 2 | 0.124 | 0.062 | 0.965 | 0.007 | 0.388 |
| RH:Season | 1 | 0.187 | 0.187 | 2.914 | 0.01 | 0.076 |
| Rainfall:RH:Season | 2 | 0.056 | 0.028 | 0.437 | 0.003 | 0.644 |
| Residuals | 132 | 8.45 | 0.064 | NA | 0.46 | NA |
| Total | 143 | 18.37 | NA | NA | 1 | NA |

**Table A38. Cover C3:C4 ratio permanova analysis pre-scarab addition.**

| **Variable** | **Df** | **SumsOfSqs** | **MeanSqs** | **F.Model** | **R^2^** | **Pr(>F)** |
| --- | --- | --- | --- | --- | --- | --- |
| Rainfall | 2 | 0.03 | 0.015 | 0.023 | 0.001 | 0.975 |
| RH | 1 | 0.1 | 0.1 | 0.152 | 0.005 | 0.682 |
| Rainfall:RH | 2 | 1.651 | 0.826 | 1.255 | 0.077 | 0.297 |
| Residuals | 30 | 19.736 | 0.658 | NA | 0.917 | NA |
| Total | 35 | 21.518 | NA | NA | 1 | NA |

**Table A39. Biomass C3:C4 ratio permanova analysis.**

| **Variable** | **Df** | **SumsOfSqs** | **MeanSqs** | **F.Model** | **R^2^** | **Pr(>F)** |
| --- | --- | --- | --- | --- | --- | --- |
| Rainfall | 2 | 3.966 | 1.983 | 8.918 | 0.131 | 0.002 |
| RH | 1 | 0.211 | 0.211 | 0.947 | 0.007 | 0.334 |
| Season | 1 | 11.302 | 11.302 | 50.822 | 0.374 | 0.001 |
| Rainfall:RH | 2 | 1.005 | 0.502 | 2.259 | 0.033 | 0.119 |
| Rainfall:Season | 2 | 0.19 | 0.095 | 0.428 | 0.006 | 0.644 |
| RH:Season | 1 | 0.1 | 0.1 | 0.451 | 0.003 | 0.494 |
| Rainfall:RH:Season | 2 | 0.071 | 0.035 | 0.159 | 0.002 | 0.85 |
| Residuals | 60 | 13.343 | 0.222 | NA | 0.442 | NA |
| Total | 71 | 30.187 | NA | NA | 1 | NA |

**Table A40. Biomass C3:C4 ratio permanova analysis pre-scarab addition.**

| **Variable** | **Df** | **SumsOfSqs** | **MeanSqs** | **F.Model** | **R^2^** | **Pr(>F)** |
| --- | --- | --- | --- | --- | --- | --- |
| Rainfall | 2 | 0.5799 | 0.28994 | 0.43766 | 0.0267 | 0.655 |
| RH | 1 | 1.0759 | 1.07594 | 1.62411 | 0.04954 | 0.213 |
| Rainfall:RH | 2 | 0.188 | 0.09401 | 0.14191 | 0.00866 | 0.869 |
| Residuals | 30 | 19.8744 | 0.66248 | 0.9151 |  |  |
| Total | 35 | 21.7182 | 1 |  |  |  |

**Table A41. Plant diversity (Shannon’s H), based on cover, permanova analysis.**

| **Variable** | **Df** | **SumsOfSqs** | **MeanSqs** | **F.Model** | **R^2^** | **Pr(>F)** |
| --- | --- | --- | --- | --- | --- | --- |
| Rainfall | 2 | 6.572 | 3.286 | 6.776 | 0.065 | 0.003 |
| RH | 1 | 0.034 | 0.034 | 0.07 | 0 | 0.792 |
| Season | 1 | 65.393 | 65.393 | 134.848 | 0.643 | 0.001 |
| Rainfall:RH | 2 | 0.11 | 0.055 | 0.113 | 0.001 | 0.9 |
| Rainfall:Season | 2 | 0.292 | 0.146 | 0.301 | 0.003 | 0.741 |
| RH:Season | 1 | 0.01 | 0.01 | 0.022 | 0 | 0.878 |
| Rainfall:RH:Season | 2 | 0.265 | 0.133 | 0.273 | 0.003 | 0.774 |
| Residuals | 60 | 29.096 | 0.485 | NA | 0.286 | NA |
| Total | 71 | 101.772 | NA | NA | 1 | NA |

**Table A42. Plant diversity (Shannon’s H), based on cover, permanova analysis pre-scarab addition.**

| **Variable** | **Df** | **SumsOfSqs** | **MeanSqs** | **F.Model** | **R^2^** | **Pr(>F)** |
| --- | --- | --- | --- | --- | --- | --- |
| Rainfall | 2 | 0.8613 | 0.43067 | 1.0726 | 0.05499 | 0.394 |
| RH | 1 | 0.9937 | 0.9937 | 2.4748 | 0.06344 | 0.119 |
| Rainfall:RH | 2 | 1.7625 | 0.88127 | 2.1948 | 0.11253 | 0.139 |
| Residuals | 30 | 12.0456 | 0.40152 | 0.76904 |  |  |
| Total | 35 | 15.6631 | 1 |  |  |  |

**Table A43. Plant diversity (Shannon’s H), based on biomass, permanova analysis.**

| **Variable** | **Df** | **SumsOfSqs** | **MeanSqs** | **F.Model** | **R^2^** | **Pr(>F)** |
| --- | --- | --- | --- | --- | --- | --- |
| Rainfall | 2 | 0.299 | 0.15 | 3.425 | 0.054 | 0.037 |
| RH | 1 | 0.02 | 0.02 | 0.462 | 0.004 | 0.48 |
| Season | 1 | 2.196 | 2.196 | 50.25 | 0.395 | 0.001 |
| Rainfall:RH | 2 | 0.023 | 0.011 | 0.258 | 0.004 | 0.762 |
| Rainfall:Season | 2 | 0.083 | 0.041 | 0.945 | 0.015 | 0.366 |
| RH:Season | 1 | 0.177 | 0.177 | 4.059 | 0.032 | 0.05 |
| Rainfall:RH:Season | 2 | 0.138 | 0.069 | 1.579 | 0.025 | 0.198 |
| Residuals | 60 | 2.623 | 0.044 | NA | 0.472 | NA |
| Total | 71 | 5.559 | NA | NA | 1 | NA |

**Table A44. Plant diversity (Shannon’s H), based on biomass, permanova analysis pre-scarab addition.**

| **Variable** | **Df** | **SumsOfSqs** | **MeanSqs** | **F.Model** | **R^2^** | **Pr(>F)** |
| --- | --- | --- | --- | --- | --- | --- |
| Rainfall | 2 | 0.08057 | 0.04028 | 0.7685 | 0.03673 | 0.474 |
| RH | 1 | 0.43783 | 0.43783 | 8.3523 | 0.19961 | 0.01 |
| Rainfall:RH | 2 | 0.1024 | 0.0512 | 0.9767 | 0.04669 | 0.402 |
| Residuals | 30 | 1.5726 | 0.05242 | 0.71697 |  |  |
| Total | 35 | 2.1934 | 1 |  |  |  |

**Table A45. Plant evenness, based on cover, permanova analysis.**

| **Variable** | **Df** | **SumsOfSqs** | **MeanSqs** | **F.Model** | **R^2^** | **Pr(>F)** |
| --- | --- | --- | --- | --- | --- | --- |
| Rainfall | 2 | 0.086 | 0.043 | 0.55 | 0.012 | 0.606 |
| RH | 1 | 0.237 | 0.237 | 3.014 | 0.033 | 0.08 |
| Season | 1 | 1.89 | 1.89 | 24.064 | 0.263 | 0.001 |
| Rainfall:RH | 2 | 0.037 | 0.019 | 0.237 | 0.005 | 0.789 |
| Rainfall:Season | 2 | 0.087 | 0.044 | 0.554 | 0.012 | 0.542 |
| RH:Season | 1 | 0.027 | 0.027 | 0.341 | 0.004 | 0.565 |
| Rainfall:RH:Season | 2 | 0.122 | 0.061 | 0.779 | 0.017 | 0.473 |
| Residuals | 60 | 4.712 | 0.079 | NA | 0.655 | NA |
| Total | 71 | 7.199 | NA | NA | 1 | NA |

**Table A46. Plant evenness, based on cover, permanova analysis pre-scarab addition.**

| **Variable** | **Df** | **SumsOfSqs** | **MeanSqs** | **F.Model** | **R^2^** | **Pr(>F)** |
| --- | --- | --- | --- | --- | --- | --- |
| Rainfall | 2 | 0.1528 | 0.076403 | 0.47713 | 0.0293 | 0.604 |
| RH | 1 | 0.221 | 0.221005 | 1.38016 | 0.04237 | 0.245 |
| Rainfall:RH | 2 | 0.038 | 0.019001 | 0.11866 | 0.00729 | 0.891 |
| Residuals | 30 | 4.8039 | 0.16013 | 0.92104 |  |  |
| Total | 35 | 5.2157 | 1 |  |  |  |

**Table A47. Plant evenness, based on biomass, permanova analysis.**

| **Variable** | **Df** | **SumsOfSqs** | **MeanSqs** | **F.Model** | **R^2^** | **Pr(>F)** |
| --- | --- | --- | --- | --- | --- | --- |
| Rainfall | 2 | 0.313 | 0.156 | 0.935 | 0.012 | 0.406 |
| RH | 1 | 0.003 | 0.003 | 0.017 | 0 | 0.881 |
| Season | 1 | 13.039 | 13.039 | 77.965 | 0.518 | 0.001 |
| Rainfall:RH | 2 | 0.284 | 0.142 | 0.848 | 0.011 | 0.45 |
| Rainfall:Season | 2 | 0.245 | 0.123 | 0.733 | 0.01 | 0.47 |
| RH:Season | 1 | 0.637 | 0.637 | 3.808 | 0.025 | 0.049 |
| Rainfall:RH:Season | 2 | 0.624 | 0.312 | 1.867 | 0.025 | 0.186 |
| Residuals | 60 | 10.034 | 0.167 | NA | 0.399 | NA |
| Total | 71 | 25.178 | NA | NA | 1 | NA |

**Table A48. Plant evenness, based on biomass, permanova analysis pre-scarab addition.**

| **Variable** | **Df** | **SumsOfSqs** | **MeanSqs** | **F.Model** | **R^2^** | **Pr(>F)** |
| --- | --- | --- | --- | --- | --- | --- |
| Rainfall | 2 | 0.0626 | 0.0313 | 0.2004 | 0.00803 | 0.837 |
| RH | 1 | 3.0216 | 3.02162 | 19.3451 | 0.38769 | 0.001 |
| Rainfall:RH | 2 | 0.0238 | 0.01189 | 0.0761 | 0.00305 | 0.925 |
| Residuals | 30 | 4.6859 | 0.1562 | 0.60122 |  |  |
| Total | 35 | 7.7939 | 1 |  |  |  |

**Table A49. Scarab extraction zero-inflated count model, with least ratio comparison with and without RH in the model.**

|  | Estimate | Std. Error | z value | Pr(>\|z\|) |  |
| --- | --- | --- | --- | --- | --- |
| **(Intercept)** | -0.8751 | 0.7014 | -1.2477 | 0.2122 | Count Model |
| **RH+** | 2.3153 | 0.752 | 3.0787 | 0.0021 |  |
| **RA** | -1.0235 | 0.449 | -2.2793 | 0.0226 |  |
| **RF** | -0.2777 | 0.6869 | -0.4043 | 0.686 |  |
| **(Intercept)** | -3.1599 | 10.7156 | -0.2949 | 0.7681 | Zero Inflated model |
| **RH+** | 3.7833 | 11.1736 | 0.3386 | 0.7349 |  |
| **RA** | -11.0919 | 157.3965 | -0.0705 | 0.9438 |  |
| **RF** | 1.0144 | 1.9205 | 0.5282 | 0.5974 |  |
|  | NA | NA | NA | NA |  |
| **#DF** | **LogLik** | **Df** | **Chisq** | **Pr(>Chisq)** |  |
| **8** | -35.8181 | NA | NA | NA | Model with RH |
| **6** | -43.3191 | -2 | 15.0018 | 0.0006 | Model without RH |

**Table A50.Per plot percentage of live biomass for the top 4 species found in all seasons.**

| **Season** | **Rainfall** | **Species** | **Mass (%)** | **SE** |
| --- | --- | --- | --- | --- |
| Cool Season 2013 | Amb | *C. dactylon* | 11.6 | 3.1 |
| Cool Season 2013 | RA | *C. dactylon* | 12.5 | 3.2 |
| Cool Season 2013 | RF | *C. dactylon* | 15.6 | 4.8 |
| Warm Season 2014 | Amb | *C. dactylon* | 5.4 | 2.1 |
| Warm Season 2014 | RA | *C. dactylon* | 13.5 | 5.5 |
| Warm Season 2014 | RF | *C. dactylon* | 4.1 | 1.1 |
| Cool Season 2014 | Amb | *C. dactylon* | 5.6 | 2.0 |
| Cool Season 2014 | RA | *C. dactylon* | 18.2 | 7.8 |
| Cool Season 2014 | RF | *C. dactylon* | 2.1 | 0.8 |
| Warm Season 2015 | Amb | *C. dactylon* | 4.1 | 2.4 |
| Warm Season 2015 | RA | *C. dactylon* | 11.7 | 4.7 |
| Warm Season 2015 | RF | *C. dactylon* | 6.7 | 3.4 |
| Cool Season 2013 | Amb | *E. curvula* | 19.1 | 4.8 |
| Cool Season 2013 | RA | *E. curvula* | 26.1 | 6.9 |
| Cool Season 2013 | RF | *E. curvula* | 27.7 | 5.4 |
| Warm Season 2014 | Amb | *E. curvula* | 6.0 | 2.3 |
| Warm Season 2014 | RA | *E. curvula* | 14.7 | 5.7 |
| Warm Season 2014 | RF | *E. curvula* | 19.5 | 8.2 |
| Cool Season 2014 | Amb | *E. curvula* | 20.5 | 6.1 |
| Cool Season 2014 | RA | *E. curvula* | 35.4 | 9.7 |
| Cool Season 2014 | RF | *E. curvula* | 40.6 | 8.4 |
| Warm Season 2015 | Amb | *E. curvula* | 12.9 | 3.3 |
| Warm Season 2015 | RA | *E. curvula* | 15.9 | 6.0 |
| Warm Season 2015 | RF | *E. curvula* | 21.3 | 5.6 |
| Cool Season 2013 | Amb | *M. stipoides* | 14.4 | 3.8 |
| Cool Season 2013 | RA | *M. stipoides* | 9.5 | 2.1 |
| Cool Season 2013 | RF | *M. stipoides* | 13.9 | 3.7 |
| Warm Season 2014 | Amb | *M. stipoides* | 7.1 | 2.4 |
| Warm Season 2014 | RA | *M. stipoides* | 2.9 | 0.9 |
| Warm Season 2014 | RF | *M. stipoides* | 8.5 | 4.0 |
| Cool Season 2014 | Amb | *M. stipoides* | 15.3 | 6.3 |
| Cool Season 2014 | RA | *M. stipoides* | 6.5 | 2.1 |
| Cool Season 2014 | RF | *M. stipoides* | 13.0 | 4.1 |
| Warm Season 2015 | Amb | *M. stipoides* | 4.9 | 2.2 |
| Warm Season 2015 | RA | *M. stipoides* | 1.8 | 0.8 |
| Warm Season 2015 | RF | *M. stipoides* | 3.7 | 1.9 |
| Cool Season 2013 | Amb | *P. dilitatum* | 16.9 | 2.3 |
| Cool Season 2013 | RA | *P. dilitatum* | 12.0 | 2.7 |
| Cool Season 2013 | RF | *P. dilitatum* | 11.5 | 2.7 |
| Warm Season 2014 | Amb | *P. dilitatum* | 1.1 | 1.1 |
| Warm Season 2014 | RA | *P. dilitatum* | 0.3 | 0.3 |
| Warm Season 2014 | RF | *P. dilitatum* | 6.9 | 4.9 |
| Cool Season 2014 | Amb | *P. dilitatum* | 27.1 | 4.6 |
| Cool Season 2014 | RA | *P. dilitatum* | 19.2 | 4.6 |
| Cool Season 2014 | RF | *P. dilitatum* | 16.2 | 4.8 |
| Warm Season 2015 | Amb | *P. dilitatum* | 33.5 | 7.0 |
| Warm Season 2015 | RA | *P. dilitatum* | 24.7 | 5.6 |
| Warm Season 2015 | RF | *P. dilitatum* | 30.7 | 8.9 |

**Figure A1. A) Soil moisture (%SWC) and simulated rain (mm) 2013-12-02 to 2015-12-02; dashed line represents mean, B) Soil moisture (%SWC) and simulated rain (mm) over the warmest portion of 1 year 2014-09-01 to 2015-01-28; dashed line represents mean.**

**
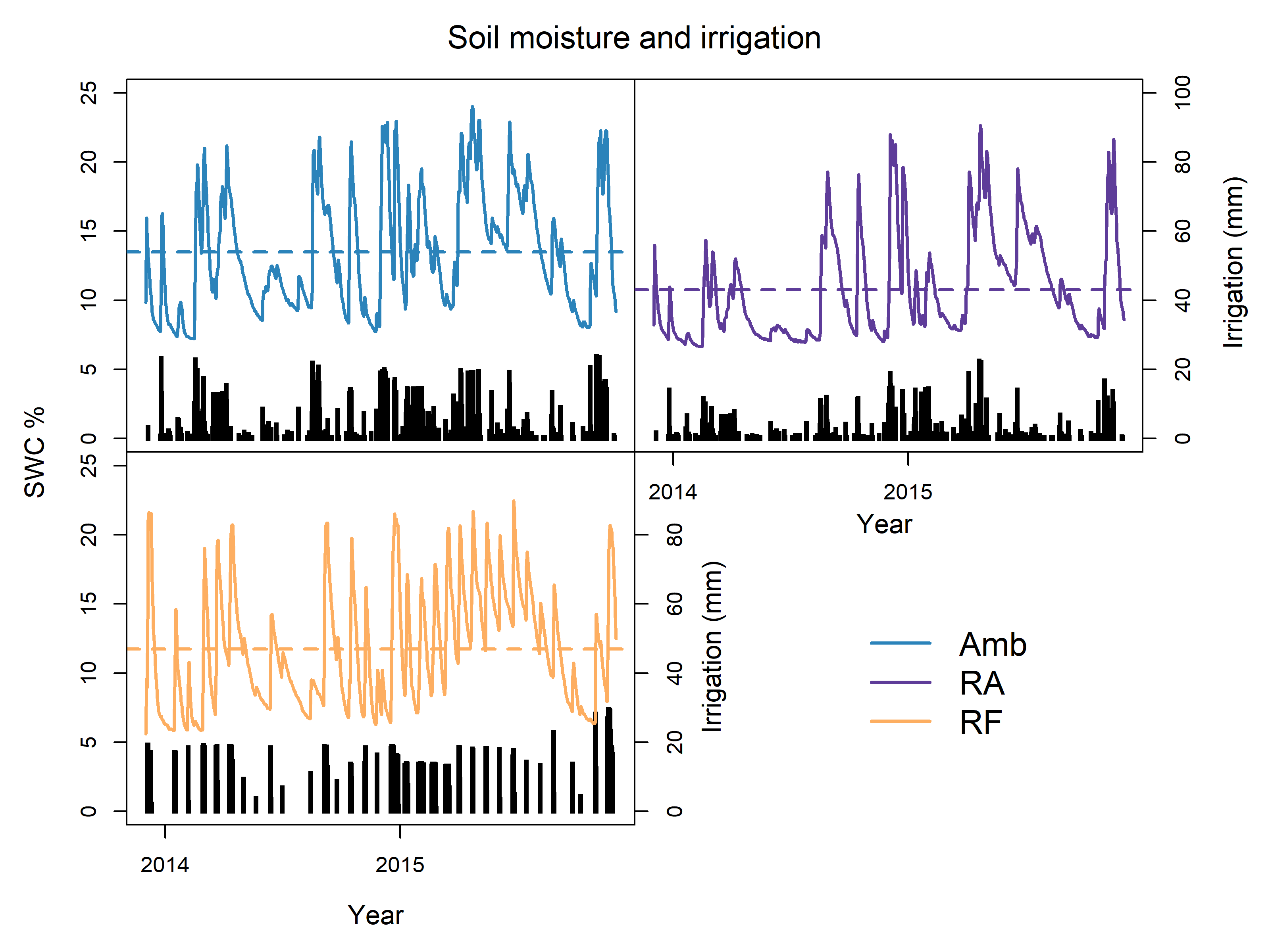

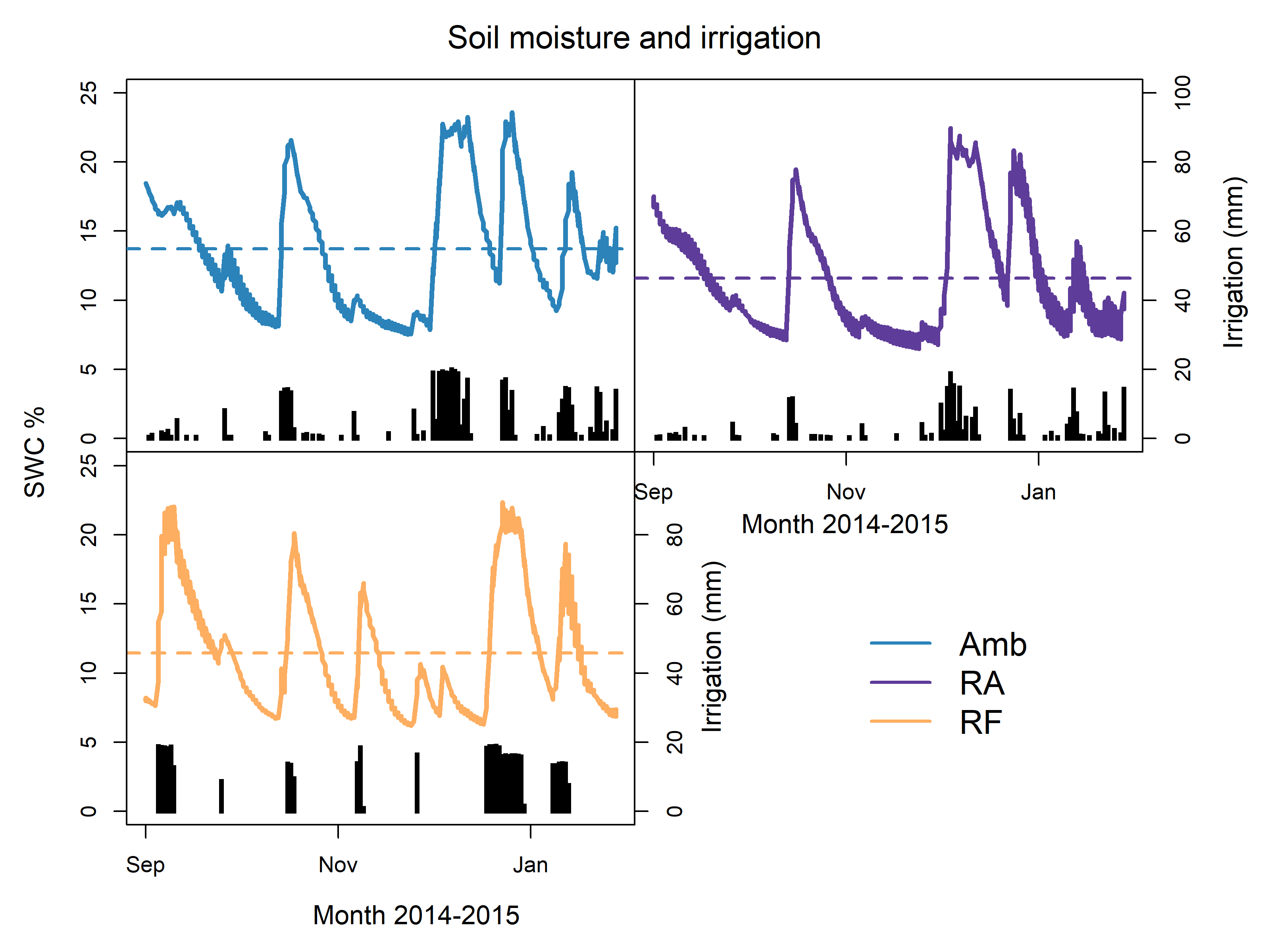
**

**B**

**A**

**Figure A2. Scaled scarab larva numbers within sub plots (Two 25 cm × 10 cm × 20 cm holes) within each grassland plot.**

**
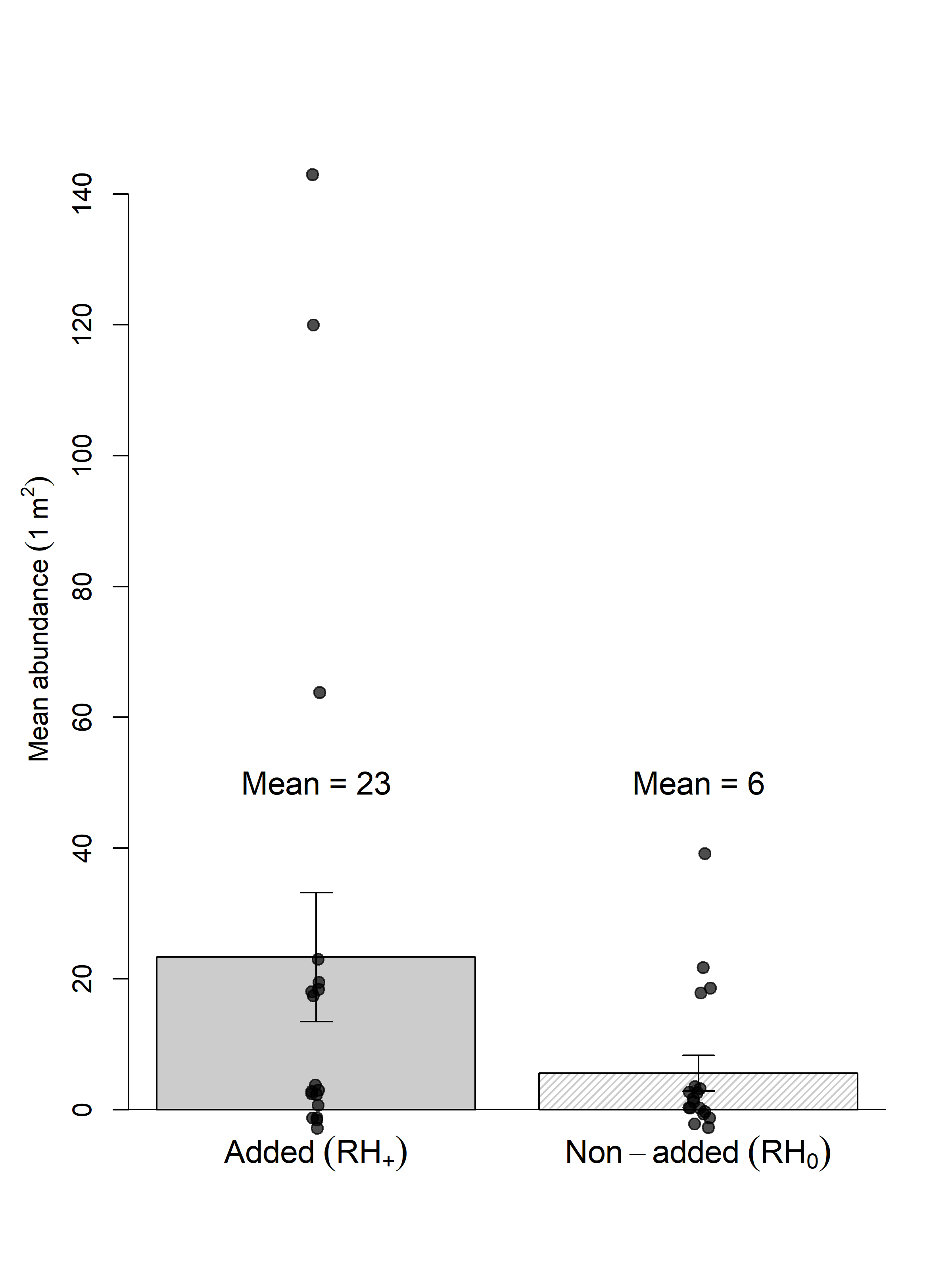
**

**Figure A3. Herbivore netting used to hold beetles within treatment plots; sides were held down with pegs. Tents were removed during peak daylight hours.**

**
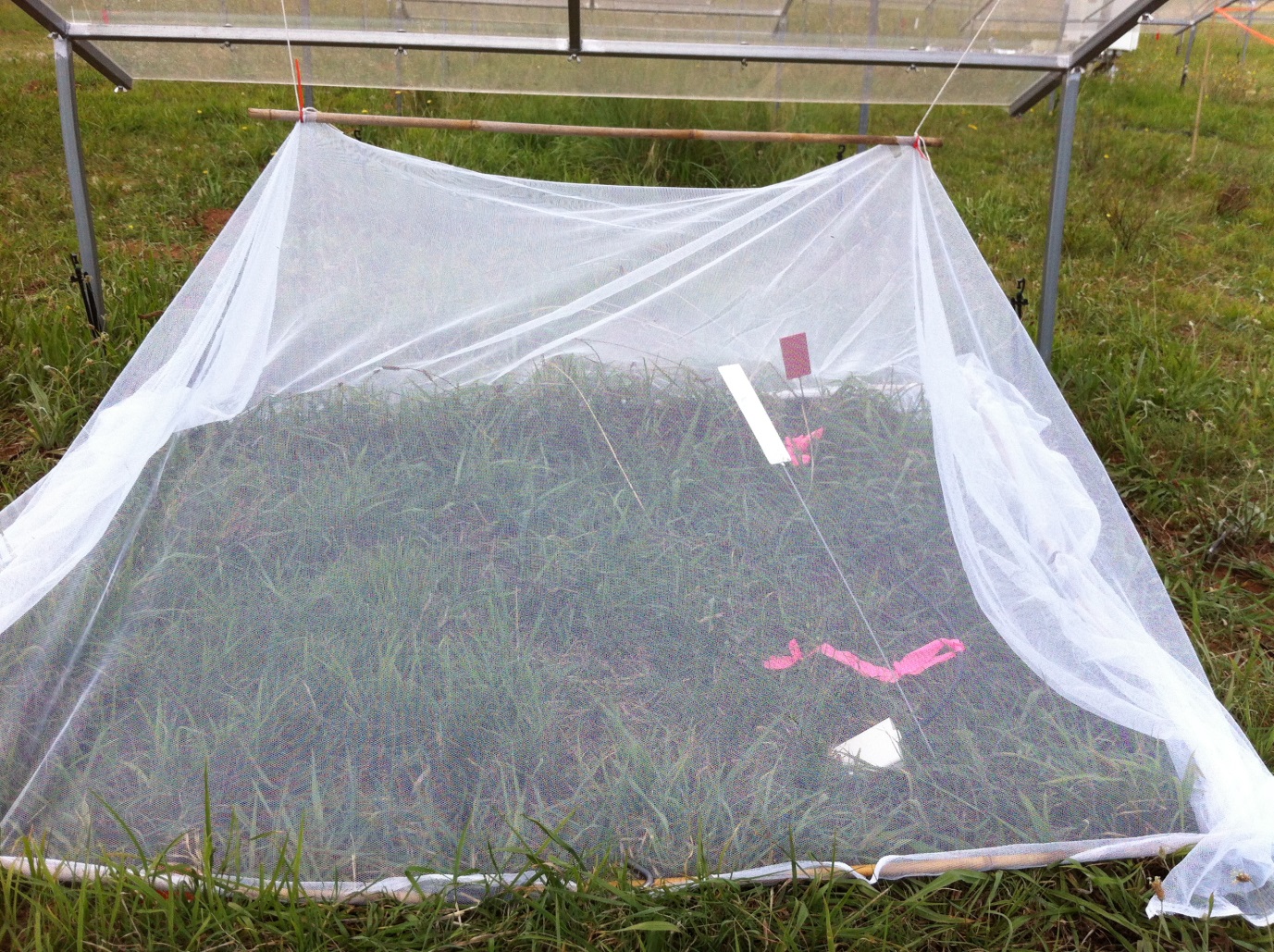
**

Figure A4. Scaled and normalized dry root mass obtained from root-ingrowth cores. RA – Reduced Amount rainfall, RF – Reduced Frequency rainfall, RH_0_ – No scarabs added, RH_+_ - Scarabs added.


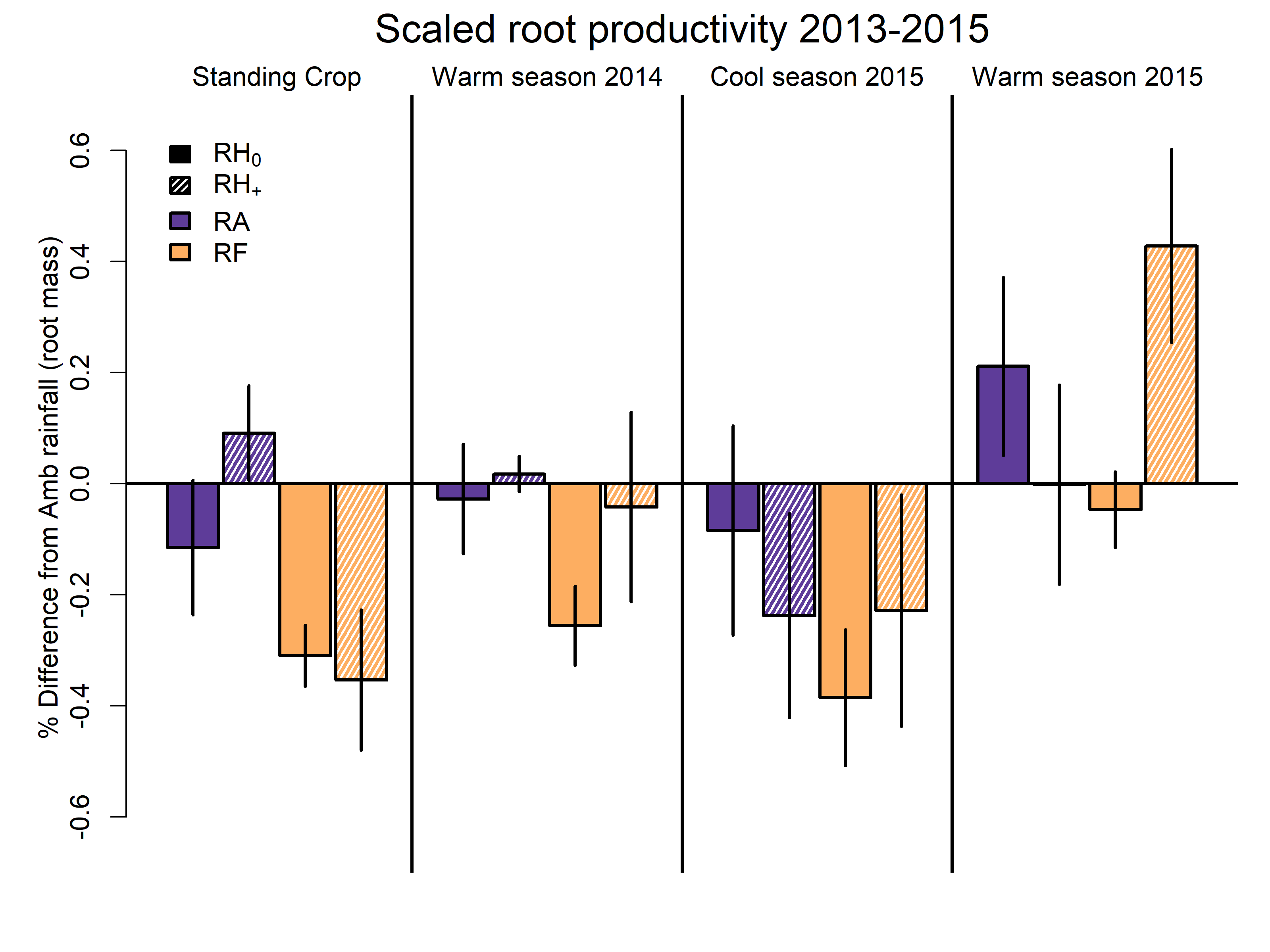

Supplement: Supplementary file 1 — Additional file 1: Table A1. Plant responses to rainfall treatments, herbivore addition, and season: cover (frequency per m2) estimates for all plots during February 2014 through August 2015 of the experiment. Table A2. Plant responses to rainfall treatments and season: cover (frequency per m2) estimates for all plots during February 2014 through August 2015 of the experiment. Table A3. Plant responses to season: cover (frequency per m2) estimates for all plots during February 2014 through August 2015 of the experiment. Table A4. Cover means over time. Table A5. Plant responses to rainfall treatments, herbivore addition, and season: dry live + dead mass (g/m2) estimates for all plots during October 2014 through April 2015 of the experiment. Table A6. Plant responses to rainfall treatments and season: dry live + dead mass (g/m2) estimates for all plots during October 2014 through April 2015 of the experiment. Table A7. Plant responses to season: dry live + dead mass (g/m2) estimates for all plots during October 2014 through April 2015 of the experiment. Table A8. Plant responses to season: dry live + dead mass (g/m2) estimates for all plots over time. Table A9. Plant responses to rainfall treatments, herbivore addition, and season: dry live mass (g/m2) estimates for all plots during October 2014 through April 2015 of the experiment. Table A10. Plant responses to rainfall treatments and season: dry live mass (g/m2) estimates for all plots during October 2014 through April 2015 of the experiment. Table A11. Plant responses to season: dry live mass (g/m2) estimates for all plots during October 2014 through April 2015 of the experiment. Table A12. Plant responses to scarab addition: dry live mass (g/m2) estimates during October 2013 (pre-scarab) through April 2015 of the experiment. Table A13. Plant responses to season: dry live (g/m2) estimates for all plots over time. Table A14. Plant responses to rainfall treatments, herbivore addition, and season: dry dead mass (g/m2 [file 12862_2021_1871_MOESM1_ESM.docx]
